# *Porphyromonas gingivalis*-derived extracellular vesicles aggravate bone destruction in rheumatoid arthritis by promoting Syk-dependent osteoclastogenesis

**DOI:** 10.1038/s41368-025-00416-1

**Published:** 2026-02-05

**Authors:** Jiajie Guo, Qiujing Qiu, Xiaoyuan Yan, Zeying Zhang, Xiyue Zhang, Na An, Chengcheng Yin, Di Yang, Hirohiko Okamura, Kaya Yoshida, Hongchen Sun, Lihong Qiu

**Affiliations:** 1 Department of Endodontics, School and Hospital of Stomatology, China Medical University, Liaoning Provincial Key Laboratory of Oral Diseases, Shenyang, China; 2 Department of Orthodontics, School and Hospital of Stomatology, China Medical University, Liaoning Provincial Key Laboratory of Oral Diseases, Shenyang, China; 3 School and Hospital of Stomatology, Jilin University, Changchun, China; 4 Jilin Provincial Key Laboratory of Tooth Development and Bone Remodeling, Changchun, China; 5 Department of Oral Morphology, Graduate School of Medicine, Dentistry and Pharmaceutical Sciences, Okayama University, Okayama, Japan; 6 Department of Oral Healthcare Education, Institute of Biomedical Sciences, Tokushima University Graduate School, Tokushima, Japan; 7 Department of Oral Pathology, School and Hospital of Stomatology, China Medical University, Liaoning Provincial Key Laboratory of Oral Diseases, Shenyang, China

**Keywords:** Mechanisms of disease, Bacterial host response

## Abstract

Rheumatoid arthritis (RA) is an autoimmune disorder that triggers progressive joint destruction by inducing excessive osteoclastogenesis. *Porphyromonas gingivalis* (*Pg*), the main pathogenic bacterium involved in periodontitis (PD), is closely related to RA. *Pg* can secrete extracellular vesicles (EVs), which carry numerous virulence factors. The aim of this study was to investigate whether *Pg-*derived EVs can be transported and exacerbate bone destruction in RA by promoting osteoclastogenesis and to elucidate the underlying mechanisms involved. EVs derived from *Porphyromonas endodontalis* (*Pe*), which is weakly associated with PD or RA, were used as controls. *Pg* and *Pe* EVs interact with osteoclasts after translocating into the marrow and metacarpal joints of mice. In vitro, *Pg* EVs induce osteoclastogenesis via various components, such as lipopolysaccharide, proteins, lipoproteins, and proteases. TNF-α, IL-1β, and IL-6 promote but cannot independently control *Pg* EV-induced osteoclastogenesis. RNA sequencing and verification experiments further demonstrated that *Pg* EVs induced osteoclastogenesis by promoting the phosphorylation of spleen tyrosine kinase (Syk). In vivo, *Pg* EVs exacerbated RA-induced bone destruction by activating Syk-dependent osteoclastogenesis. R406, a Syk inhibitor, significantly attenuated *Pg* EV-induced RA osteoclastogenesis and bone destruction. However, *Pe-*derived EVs presented an extremely weak ability to promote osteoclastogenesis and RA. Our findings reveal a new mechanism by which *Pg* EVs can exacerbate RA via transport through the circulation and the promote Syk-dependent osteoclastogenesis. This study deepens our understanding of the significant pathogenic role of EVs derived from oral bacterial in RA and explores targeted therapeutic strategies by inhibiting the activation of Syk.

## Introduction

Periodontitis (PD) and systemic diseases have recently received widespread attention. Rheumatoid arthritis (RA) is a systemic inflammatory autoimmune disease characterized by synovitis, progressive destruction of bone and cartilage, and joint dysfunction that affects nearly 1%–2% of the population worldwide.^1^ Research has emphasized the bidirectional relationship between PD and RA.^2–4^ *Porphyromonas gingivalis* (*Pg*), a black-pigmented gram-negative anaerobe, is the main pathogenic bacteria involved in PD.^5^ *Pg* is strongly associated with RA because it triggers a citrulline-specific autoimmune response,^6^ activates complement C5a,^7^ and leads to proinflammatory cytokine overexpression.^8^ Thus, *Pg* affects the progression of RA through indirect immune effects. However, while DNA components of *Pg* are detectable in the serum or synovial fluid of patients with RA, it is difficult to verify the presence of live *Pg* via bacterial culture.^9,10^ Thus, components of *Pg* may be transported and act directly on joint tissues. However, research on this mechanism is currently lacking.

*Pg* can secrete nanometer-sized extracellular vesicles (EVs) through outer membrane budding.^11^ EVs carry numerous types of cargo from their parent bacteria, including lipoproteins, lipopolysaccharide (LPS), periplasm, nucleic acids, and cellular contents, which are either embedded in the phospholipid bilayer or wrapped in the vesicles.^12^ EVs from oral bacteria can transport assembled cargos to distant sites and evade immune clearance, thus mediating systemic disease. Studies have revealed that EVs from oral pathogenic bacteria can be transported to organs such as the liver and brain, thereby altering glucose metabolism or causing neuroinflammation.^13,14^ These studies led us to explore whether *Pg*-derived EVs can be transferred to the bone marrow or joints, exacerbating bone and joint damage in patients with RA.

Active osteoclastogenesis is a major cause of bone and joint destruction.^15^ Osteoclasts are multinucleated cells formed by the fusion of mononuclear cells. Receptor activator of nuclear factor-κB ligand (RANKL) binds to the precursor osteoclast membrane receptor RANK and activates the transcription factor NFATc1 to promote osteoclastogenesis via the classical signaling pathway.^16^ Although researchers have proposed that *Pg*, LPS, lipids, or fimbriae can promote osteoclastogenesis, the specific cellular and biological mechanisms involved remain to be fully elucidated.^17–19^ Most importantly, whether *Pg*-derived EVs are more effective at promoting osteoclastogenesis than these individual virulence factors has not been thoroughly investigated.

Here, we focused on osteoclastogenesis. To eliminate potential experimental bias caused by nonspecific immune responses to bacteria, we used EVs derived from *Porphyromonas endodontalis* (*Pe*) as a control. *Pe* belongs to the same genus as *Pg* but lacks several powerful virulence factors. *Pe* also shows weaker correlations with PD and RA.^20,21^ In this study, we intended to investigate (i) whether *Pg* EVs and *Pe* EVs can be transported to joints and bone marrow cavities via the blood circulation; (ii) whether *Pg* EVs and *Pe* EVs can induce osteoclastogenesis and the signaling pathways involved; and (iii) whether *Pg* EVs and *Pe* EVs affect joint and bone destruction in mice with collagen-induced arthritis. The present study provides new insights into the ability of *Pg-*derived EVs to promote osteoclastogenesis and RA-related bone loss and explores targeted therapeutic strategies aimed at alleviating bone destruction in RA patients with periodontal disease.

## Results

### EVs are recruited to the bone marrow, joints and paws of mice

**Fig. 1:**
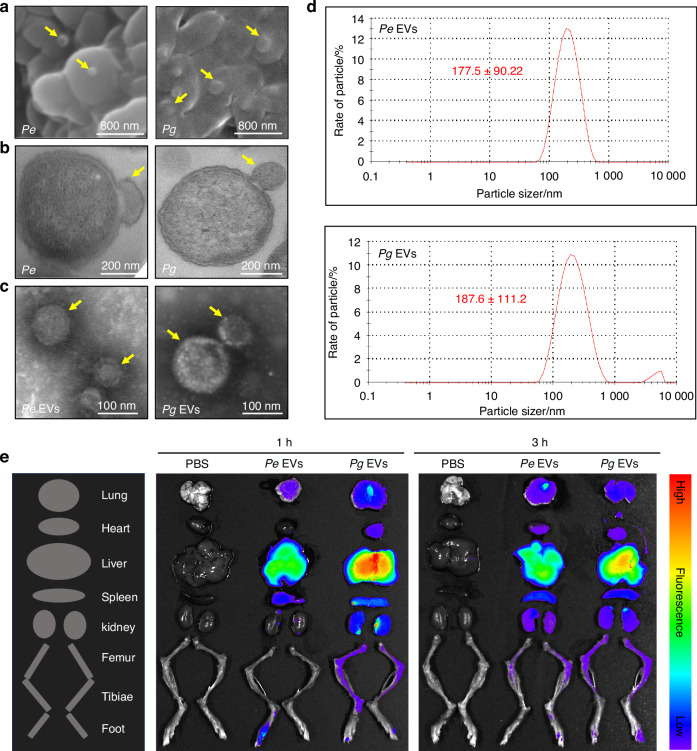
EVs are recruited to the bone marrow and paws of mice. **a**, **b** *Pe* and *Pg* were examined via SEM and transmission electron microscopy (TEM), respectively. **c** *Pe*- and *Pg*-derived EVs were examined via TEM. **d** Diameter analysis of *Pe* and *Pg* EVs. **e** DiR-labeled EV tracking in mice

We first characterized *Pg* and *Pe* and the EVs they secreted. Scanning electron microscopy (SEM) images revealed that *Pg* and *Pe* cells had bulbous vesicle protrusions from the membrane surface (Fig. 1a). The outer membranes of *Pg* and *Pe* bulged into a spherical vesicle with a diameter of 150–200 nm (Fig. 1b). The *Pg* and *Pe* EVs had single-membrane vesicular structures without residual bacterial cells (Fig. 1c). The diameter of the EVs was measured via Nanoparticle tracking analysis (NTA). The mean size of the *Pe*-derived EVs was 177.5 nm, and that of the *Pg*-derived EVs was 187.6 nm (Fig. 1d). Silver staining revealed that many EV protein bands of *Pg* and *Pe* corresponded to those of the bacterial cells (Fig. S1). These results suggest that we successfully isolated EVs from *Pg* and *Pe* and that the isolated *Pg* and *Pe* EVs had intact vesicle structures.

To examine whether *Pg* and *Pe* EVs can translocate to distant organs in vivo, DiR-labeled EVs were injected intraperitoneally into mice. The results showed that *Pg* and *Pe* EVs can be transported to the lung, heart, liver, spleen, kidneys, long bone marrow, joints, and paws within 3 h. *Pg* and *Pe* EVs accumulated most strongly in the liver at 1 h, but their accumulation in the liver decreased at 3 h and increased in the bone marrow. Moreover, compared with *Pe* EVs, *Pg* EVs presented greater transport efficiency in all organs (Fig. 1e). Together, these results demonstrate that *Pg* and *Pe* EVs can be transported to the bone marrow and paws in vivo.

### *Pg-*derived EVs induce osteoclastogenesis in vitro

**Fig. 2:**
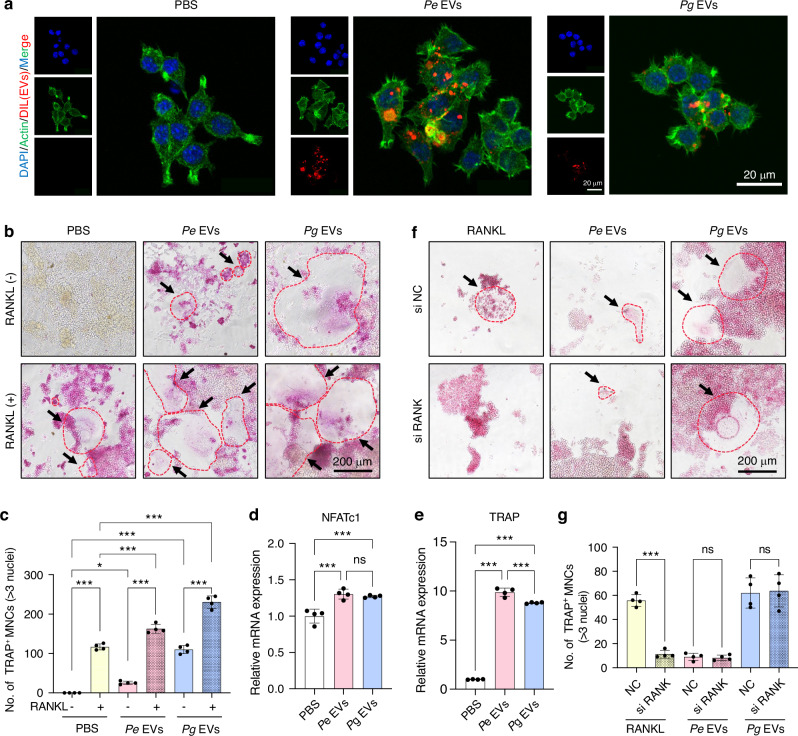
EVs induce osteoclastogenesis in vitro. **a** Fluorescent localization of DIL-labeled EVs in RAW264.7 cells. **b** RAW264.7 cells were induced with EVs with or without RANKL for 6 days, and TRAP staining was performed. **c** TRAP-positive multinuclear cells with more than 3 nuclei were counted. **d**, **e** RAW264.7 cells were treated with EVs for 48 h, and the expression of osteoclast generation marker genes was measured by RT‒PCR. **f** RAW264.7 cells were treated with EVs and RANKL for 6 days with or without RANK siRNA (siRANK), and TRAP staining was performed. **g** TRAP-positive multinuclear cells with more than 3 nuclei were counted. ns *P* > 0.05, ^*^P < 0.05, ^***^P < 0.001. The red dashed line and arrows represent TRAP-positive osteoclasts

The monocyte/macrophage-like RAW264.7 cells were subsequently stimulated with EVs. We referred to studies on the effects of EVs secreted by *Pg* and *Fusobacterium nucleatum* on macrophages and after comprehensively considering the stability of *Pg* EVs in inducing osteoclastogenesis selected an EV concentration of 1 μg/mL for in vitro experiments.^22,23^ EVs labeled with DIL could be internalized by RAW264.7 cells in a short time (Fig. 2a). Next, RAW264.7 cells were treated with EVs with or without RANKL. TRAP staining (Fig. 2b) and merged images of F-actin and nuclei (Fig. S2) revealed that both *Pg* and *Pe* EVs promoted osteoclastogenesis regardless of the presence or absence of RANKL. However, the size and number of osteoclasts induced by *Pg* EVs were greater than those induced by *Pe* EVs (Fig. 2b, c). The gene expression of osteoclast markers was also increased by *Pg* and *Pe* EVs (Figs. 2d, e and S3). RANK was subsequently silenced in RAW264.7 cells via siRANK. The inhibition efficiency of RANK was confirmed via RT‒qPCR (Fig. S4). Knockdown of RANK markedly inhibited RANKL-induced osteoclastogenesis but had no inhibitory effect on *Pg* EV- or *Pe* EV-triggered osteoclastogenesis (Figs. 2f, g and S5). Similar results were obtained when osteoprotegerin (OPG), a decoy receptor for RANKL, was used (Fig. S6). After RANK was knocked down, osteoclast marker gene expression showed almost no change with *Pg* EV or *Pe* EV treatment (Fig. S7). Together, these results demonstrate that *Pg* and *Pe* EVs regulate osteoclastogenesis with or without RANKL involvement in vitro.

### *Pg* EVs mediate osteoclastogenesis through multiple active components

**Fig. 3:**
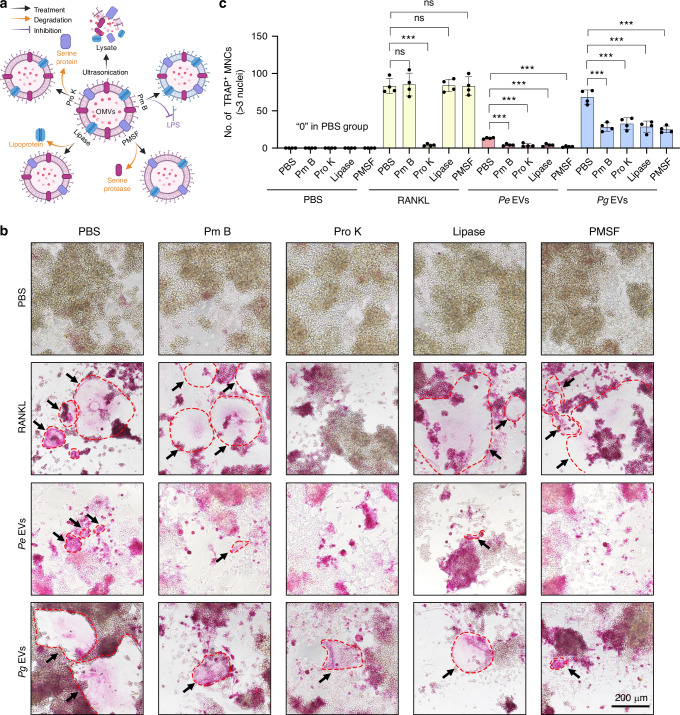
Different treatments affect *Pg* EV-induced osteoclastogenesis. **a** Schematic diagram of the different physical and biological treatments applied to EVs. The vesicular structure of EVs is disrupted by ultrasound, and the LPS, serine proteins, lipoproteins, and serine proteases in the EVs are removed or degraded by pretreatment with Pm B, Pro K, lipase, and PMSF, respectively. **b** EVs or RANKL were pretreated with Pm B (0.1 μg/μL), Pro K (0.1 μg/μL), lipase (0.1 μg/μL) and PMSF (5 mM) for 1 h at RT and then incubated at 4 °C. RAW264.7 cells were subsequently treated with Pm B-, Pro K-, lipase-, and PMSF-pretreated EVs or RANKL for 6 days, after which TRAP staining was performed. The red dashed line represents TRAP-positive osteoclasts. **c** TRAP-positive multinuclear cells with more than 3 nuclei were counted. ns *P* > 0.05, ^***^P < 0.001

To explore which components of EVs induce osteoclastogenesis, we subjected EVs to a series of treatments (Fig. 3a). Interestingly, after ultrasonic fragmentation, the *Pe* EV lysate seemed to intensify TRAP staining. However, there was a significant decrease in osteoclastogenesis induced by *Pg* EV lysates compared with that induced by intact *Pg* EVs (Fig. S8). Polymyxin B (Pm B), proteinase K (Pro K), lipoprotein lipase, and phenylmethanesulfonyl fluoride (PMSF) were subsequently used to disrupt or degrade LPS, serine protein, lipoprotein, and serine protease, respectively, in EVs (Fig. 3a). Pro K was able to degrade the recombinant protein RANKL and thus completely inhibited RANKL-induced osteoclastogenesis, whereas Pm B, lipase and PMSF had no effect on RANKL (Figs. 3b, c and S9). Pm B, Pro K, lipase, and PMSF inhibited the formation of osteoclasts induced by *Pg* and *Pe* EVs to varying degrees, with PMSF resulting in a stronger inhibitory effect (Figs. 3b, c and S9). These results indicate that the generation of osteoclasts induced by EVs, especially *Pg* EVs, depends on the structural integrity of the EVs and the presence of multiple active components, such as LPS, proteins, lipoproteins, and serine proteases.

### TNF-α, IL-1β and IL-6 are involved in *Pg* EV-mediated osteoclastogenesis

**Fig. 4:**
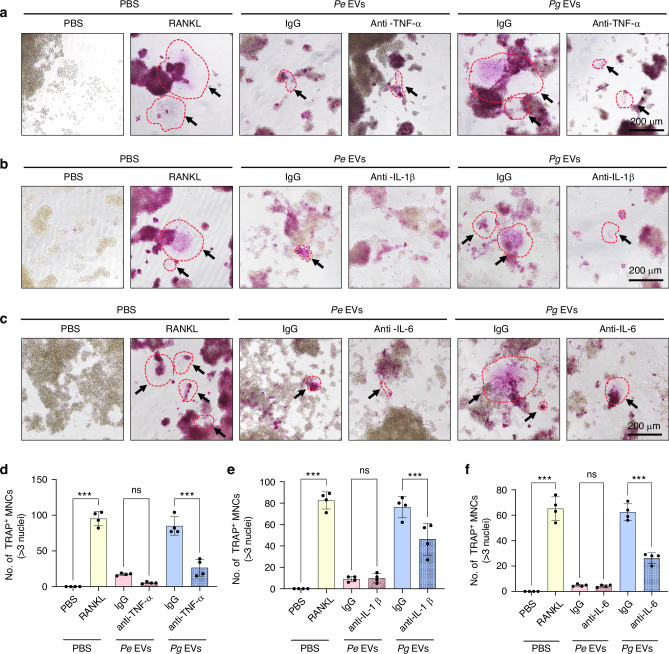
TNF-α, IL-1β and IL-6 are involved in *Pg* EV-mediated osteoclastogenesis. RAW264.7 cells were treated with EVs in the presence of neutralizing antibodies against TNF-α, IL-1β, IL-6 or IgG (2 μg/mL) for 6 days, and RANKL was used as a positive control. **a**–**c** TRAP staining was performed. **d**–**f** TRAP-positive multinuclear cells with more than 3 nuclei were counted. ns *P* > 0.05, ^***^P < 0.001. The red dashed line and arrows represent TRAP-positive osteoclasts

To further investigate whether the cytokines released from EV-stimulated RAW264.7 cells are involved in promoting osteoclastogenesis, the gene expression of TNF-α, IL-1β and IL-6 was evaluated. Both *Pg* and *Pe* EVs promoted TNF-α, IL-1β and IL-6 mRNA expression (Fig. S10). The use of neutralizing antibodies significantly reduced the levels of TNF-α, IL-1β, and IL-6 in the culture supernatants of RAW264.7 cells treated with *Pg* and *Pe* EVs (Fig. S11). As expected, neutralizing antibodies against TNF-α (Figs. 4a, d and S12a), IL-1β (Figs. 4b, e and S12b) and IL-6 (Figs. 4c, f and S12c) partially inhibited osteoclastogenesis induced by *Pg* EVs to varying degrees. However, no difference in osteoclastogenesis induced by *Pe-*derived EVs was detected after the use of neutralizing antibodies (Figs. 4 and S12). These results suggest that TNF-α, IL-1β and IL-6 facilitate *Pg* EV-mediated osteoclastogenesis.

### TNF-α, IL-1β and IL-6 cannot independently control osteoclastogenesis in *Pg* EV-stimulated RAW264.7 cells

**Fig. 5:**
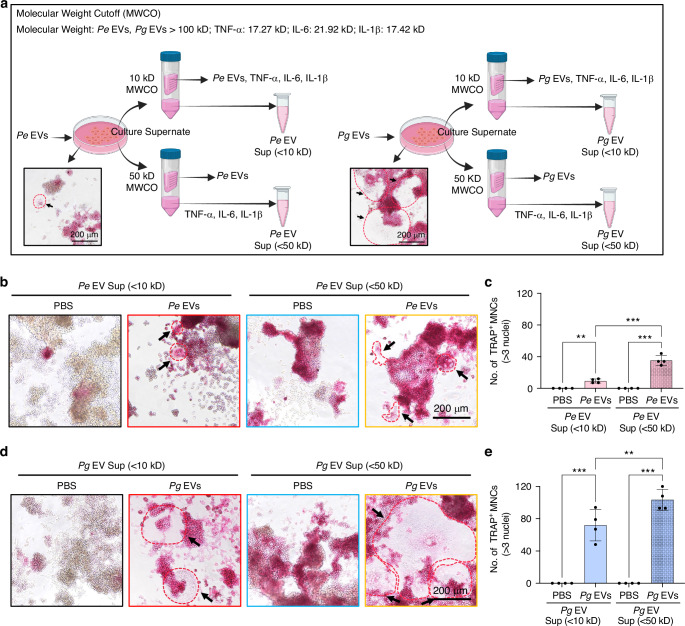
TNF-α, IL-1β and IL-6 promoted but did not independently control osteoclastogenesis in *Pg* EV-stimulated RAW264.7 cells. **a** Schematic diagram showing cell culture supernatants containing or lacking individual cytokines (TNF-α, IL-1β, or IL-6). RAW264.7 cells were treated with EVs for 2, 4, or 6 days, and the cell culture supernatant was collected at each time point. The supernatant was subsequently separated in ultrafiltration centrifuge tubes with different pore sizes to remove EVs, and supernatants containing TNF-α, IL-1β, and IL-6 (Sup < 50 kD) or lacking TNF-α, IL-1β, and IL-6 (Sup < 10 kD) were obtained. **b**, **d** Newly seeded RAW264.7 cells were cultured in the supernatant (Sup < 50 kDa or Sup < 10 kD) supplemented with fresh EVs or not for 6 days, after which TRAP staining was performed. **c**, **e** TRAP-positive multinuclear cells with more than 3 nuclei were counted. ns *P* > 0.05, ^**^P < 0.01, ^***^P < 0.001. The red dashed line and arrows represent TRAP-positive osteoclasts

To further elucidate whether EVs induce osteoclastogenesis entirely through TNF-α, IL-1β, and IL-6, we prepared culture supernatants (RAW264.7 cells stimulated with *Pe* EVs or *Pg* EVs) containing (Sup < 50 kD) or lacking (Sup < 10 kD) TNF-α, IL-1β, and IL-6 while excluding EVs (Fig. 5a). The ELISA results revealed that the levels of TNF-α, IL-1β and IL-6 in the supernatant passed through the 10 kD filter (Sup < 10 kD) were significantly lower than those in the supernatant passed through the 50 kDa filter (Sup < 50 kD) (Fig. S13). The supernatant containing TNF-α, IL-1β, and IL-6 but not containing EVs (Sup < 50 kD) had no ability to promote osteoclastogenesis (Figs. 5b‒e and S14, blue border images). However, the supernatant containing EVs but lacking TNF-α, IL-1β, and IL-6 (supernatant (Sup < 10 kD) of the EV mixture) still promoted osteoclastogenesis (Figs. 5b‒e and S14, red border images). More importantly, compared with the supernatant lacking TNF-α, IL-1β, and IL-6 (Figs. 5b‒e and S14, red border images), the supernatant containing TNF-α, IL-1β, and IL-6 exhibited a greater ability to promote TRAP-positive multinucleated cell generation in RAW264.7 cells treated with *Pe* EVs or *Pg* EVs (Figs. 5b‒e and S14, yellow border images). These results illustrate that TNF-α, IL-1β, and IL-6 indirectly promoted but did not independently control osteoclastogenesis in EV-stimulated RAW264.7 cells. Hence, EV-induced osteoclastogenesis requires the involvement of other signaling pathways rather than relying entirely on cytokines.

### *Pg* EVs induce osteoclastogenesis by activating Syk

**Fig. 6:**
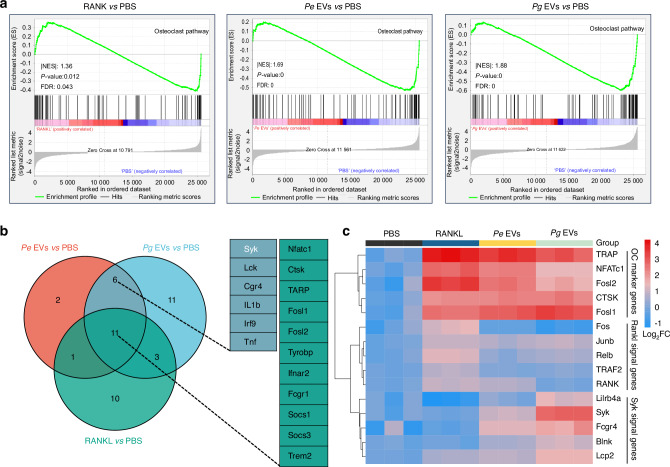
*Pg* EVs induce osteoclastogenesis by activating Syk. Transcriptomic analysis of EVs in regulating osteoclastogenesis. **a** Gene set enrichment analysis (GSEA) showing the enrichment of genes involved in the osteoclast pathway activated by RANKL and EVs. **b** Venn diagram indicating the genes upregulated during osteoclastogenesis induced by RANKL or EVs. **c** Heatmap analysis was used to analyze the expression of osteoclast (OC) marker genes, RANKL signaling-related genes and Syk signaling-related genes induced by RANKL or EVs

To further explore the mechanism of osteoclastogenesis mediated by *Pg* and *Pe* EVs, we conducted transcriptome analysis. There were 1 240 and 611 upregulated genes in the *Pg* EV and *Pe* EV groups, respectively (Fig. S15). The signaling pathways associated with osteoclast differentiation were significantly enriched in the RANKL, *Pe* EV and *Pg* EV groups (Fig. S16). The GSEA results revealed that the osteoclast signaling pathway was significantly enriched in the RANKL, *Pe* EV and *Pg* EV groups (Fig. 6a). We subsequently analyzed the upregulated genes related to osteoclast differentiation via a Venn diagram and found that the expression of 6 osteoclast differentiation-related genes was significantly increased in the *Pe* EV and *Pg* EV groups but remained unchanged in the RANKL group, with Syk being the major gene (Fig. 6b). Heatmap analysis further revealed that Syk signaling-related genes, rather than RANKL signaling-related genes, were significantly upregulated in the *Pe* EV and *Pg* EV groups (Fig. 6c).

**Fig. 7:**
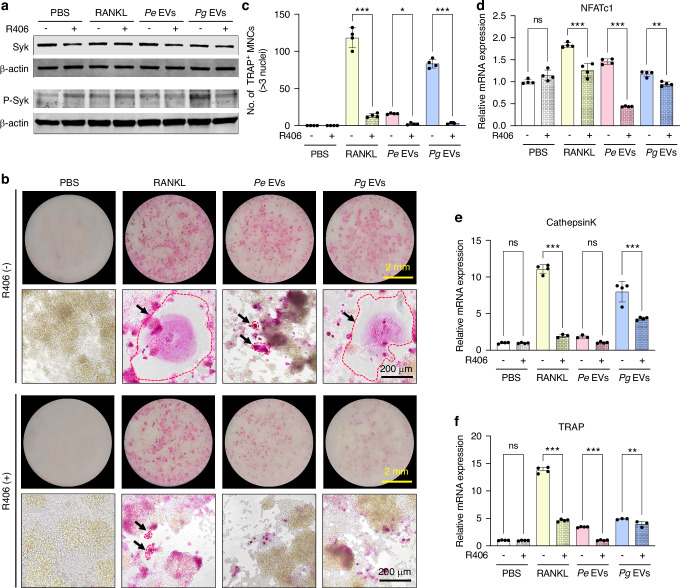
Inhibition of Syk attenuates the pro-osteoclastogenic capacity of *Pg* EVs in vitro. **a** RAW264.7 cells were treated with RANKL and EVs in the presence or absence of R406 (0.1 μmol/L, a highly selective small-molecule inhibitor of Syk) for 24 h, and Syk and p-Syk protein expression was detected via western blotting. **b** RAW264.7 cells were treated with RANKL or EVs in the presence or absence of R406 for 6 days, after which TRAP staining was performed. TRAP staining images of the stained wells of each group were captured (upper plot). TRAP staining was observed under a microscope (bottom plot). **c** TRAP-positive multinuclear cells with more than 3 nuclei were counted. **d**‒**f** RAW264.7 cells were treated with RANKL and EVs in the presence or absence of R406 for 24 h, after which the relative expression of the osteoclast generation marker genes NFATc1, Cathepsin K and TRAP was analyzed. ns *P* > 0.05, ^*^P < 0.05, ^**^P < 0.01, ^***^P < 0.001. The red dashed line and arrows represent TRAP-positive osteoclasts

R406 is a selective inhibitor of Syk. The results of the CCK-8 assay revealed that 0.1 μmol/L R406 did not affect the viability of RAW264.7 cells (Fig. S17); therefore, this concentration was used for subsequent experiments. Syk and p-Syk protein expression induced by *Pe* EVs and *Pg* EVs was inhibited by R406 to varying degrees (Figs. 7a and S18). R406 significantly inhibited the osteoclastogenesis induced by *Pe* and *Pg* EVs (Fig. 7b, c). Interestingly, R406 also significantly inhibited RANKL-induced osteoclastogenesis (Figs. 7b, c and S19). The mRNA expression of NFATc1, Cathepsin K, and TRAP in the *Pe*-EV and *Pg*-EV groups was also inhibited by R406 (Fig. 7d‒f). These results indicate that Syk activation is an important mechanism by which *Pe* EVs and *Pg* EVs promote osteoclastogenesis.

### *Pg* EVs exacerbate bone destruction in mice with RA by activating Syk

**Fig. 8:**
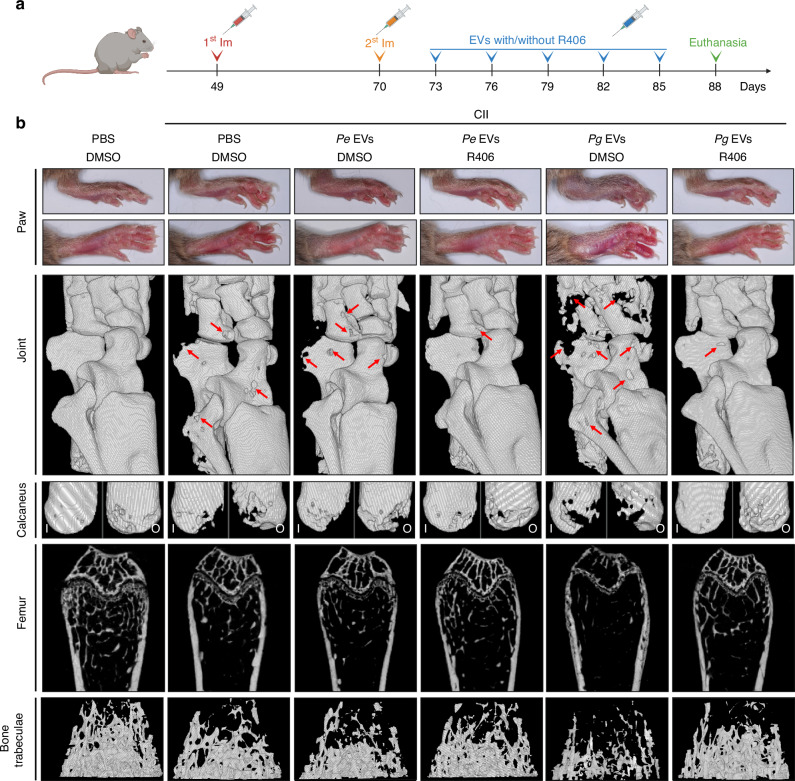
R406 alleviated RA-induced bone destruction exacerbated by *Pg-*EVs. **a** Schematic diagram of RA mice that received EVs with or without R406 administration. “Im” indicates immunization. **b** From top to bottom are shown macroscopic photos of the mouse paws, 3D reconstruction photos of the mouse ankle and metacarpal joints, 3D reconstruction images of the calcaneus, CT images of the femur, and 3D reconstruction images of the femoral bone trabeculae. Red arrows represent bone destruction. “I” represents the inner surface; “O” represents the outer surface

After we established a mouse model of RA, we coinjected R406 intraperitoneally with EVs (Fig. 8a). The rate of increase in the body weight of the RA (CII/PBS/DMSO) mice was lower than that of the healthy mice in the PBS control group (PBS/DMSO), whether they were injected with or without EVs or R406 (Fig. S20). The scores of the RA mice injected with *Pg* EVs (CII/*Pg* EVs/DMSO) were significantly greater than those of the RA mice (CII/PBS/DMSO), whereas there was no significant difference in the scores of the RA mice with (CII/*Pe* EVs/DMSO) or without *Pe* EV injection (Fig. S21). RA mice exhibited visible splenomegaly, and the injection of *Pe* and *Pg* EVs further promoted splenomegaly (Fig. S22). R406 significantly reduced the scores and splenomegaly of mice with RA injected with *Pe* EVs (CII/*Pe* EVs/R406) or *Pg* EVs (CII/*Pg* EVs/R406) to different degrees (Figs. S21 and S22). The TNF-α, IL-1β, and IL-6 levels in the serum of RA mice were elevated by treatment with *Pe* EVs or *Pg* EVs but reduced by cotreatment with R406 (Fig. S23). Arthritis was aggravated by *Pg* EVs but not by *Pe* EVs, as evidenced by indicators such as paw swelling, joint destruction, calcaneus destruction, femur osteoporosis and trabecular density (Fig. 8b). Similar trends were observed in the statistical analysis of the calcaneus bone volume, femoral bone volume fraction, and femoral bone trabecular number (Fig. S24). R406 significantly attenuated the ability of *Pg* EVs to promote arthritis (Figs. 8b and S24). The analysis of cortical bone thickness in each group did not reveal differences (Fig. S25).

### R406 alleviates *Pg* EV-induced osteoclastogenesis and Syk activation in RA mice

**Fig. 9:**
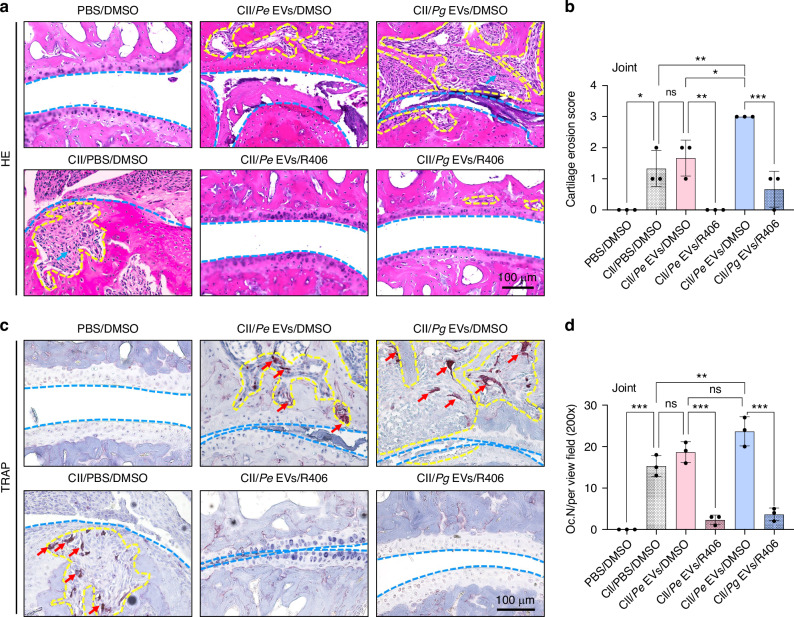
R406 attenuated *Pg* EV-induced cartilage erosion and osteoclastogenesis in the metacarpal joints of RA model mice. **a** H&E staining of the metacarpal joints. **b** Scoring of cartilage erosion in arthritic joints was evaluated according to the “SMASH” recommendations. **c** TRAP staining of osteoclasts in the metacarpal joints. **d** The number of TRAP-positive osteoclasts per field of view in the metacarpal joints. The yellow dashed line represents bone or cartilage destruction. The blue dashed line represents the articular surface. The blue arrows represent immune cell infiltration. The red arrows represent osteoclasts. ns *P* > 0.05,^***^P < 0.001

**Fig. 10:**
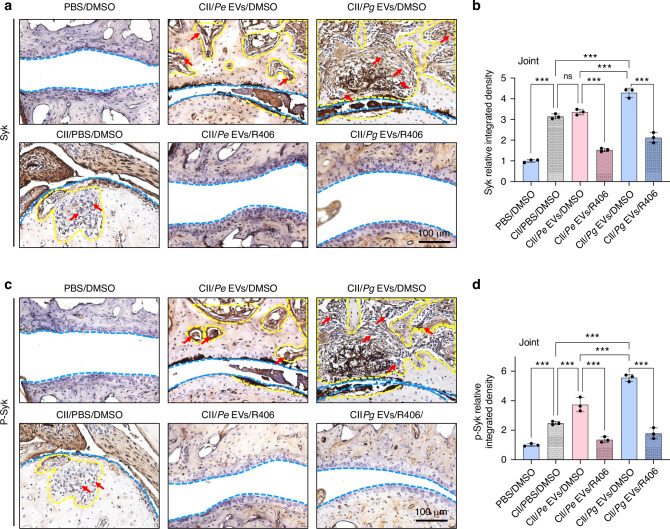
R406 inhibited *Pg* EV-promoted Syk and p-Syk expression in the metacarpal joints of RA model mice. **a** Syk staining of the metacarpal joints. **b** Relative integrated density of Syk-positive areas per view field in the metacarpal joints. **c** p-Syk staining of the metacarpal joints. **d** Relative integrated density of p-Syk-positive areas per view field in the metacarpal joints. The yellow dashed line represents bone or cartilage destruction. The blue dashed line represents the articular surface. The red arrows represent osteoclasts. ns *P* > 0.05, ^***^P < 0.001

Histological observation and analysis revealed that *Pg* EVs but not *Pe* EVs promoted bone destruction (Fig. 9a) and cartilage erosion score (Fig. 9b), osteoclastogenesis (Fig. 9c, d), Syk expression (Fig. 10a, b) and p-Syk expression (Fig. 10c, d) in the metacarpal joints of RA mice compared to those in the RA group. R406 effectively inhibited bone destruction, osteoclastogenesis, and Syk and p-Syk expression mediated by *Pg-*derived EVs and *Pe-*derived EVs in the metacarpal joint (Figs. 9 and 10).

**Fig. 11:**
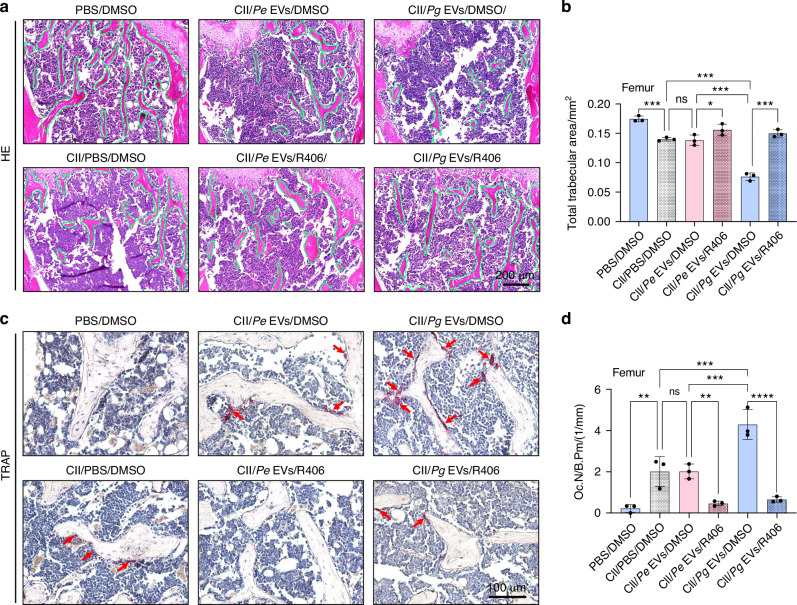
R406 attenuated *Pg* EV-induced trabecular destruction and osteoclastogenesis in the femur of RA model mice. **a** H&E staining of the femur. **b** Quantification of total trabecular bone area. **c** TRAP staining of osteoclasts in the femur. **d** The number of TRAP-positive osteoclasts per field of view in the femur. The green dashed line represents the trabeculae. Red arrows represent osteoclasts. ns *P* > 0.05, ^*^P < 0.05, ^**^P < 0.01, ^***^P < 0.001

**Fig. 12:**
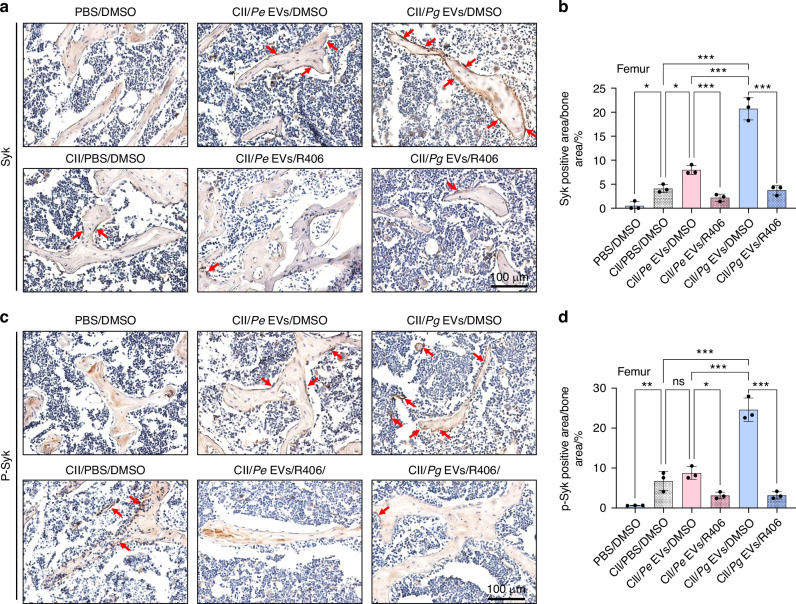
R406 inhibited *Pg* EV-promoted Syk and p-Syk expression in the femur of RA model mice. **a** Syk staining of the femur. **b** Quantification of the relative percentages of Syk-positive areas in the femur. **c** p-Syk staining of the femur. **d** Quantification of the relative percentages of p-Syk-positive areas in the femur. The red arrows represent osteoclasts. ns *P* > 0.05, ^*^P < 0.05, ^**^P < 0.01, ^***^P < 0.001

Similar trends were observed in the femur. *Pg* EVs but not *Pe* EVs further reduced the trabecular bone area in RA mice (Fig. 11a, b), leading to osteoporosis, accompanied by increased osteoclastogenesis around trabeculae (Fig. 11c, d) and significant upregulation of Syk (Fig. 12a, b) and p-Syk expression (Fig. 12c, d). R406 inhibited osteoclastogenesis by reducing the expression of Syk and p-Syk, thereby significantly attenuating femoral osteoporosis in mice with RA aggravated by *Pg* EVs (Figs. 11 and 12).

Overall, *Pg-*derived EVs exacerbated joint destruction and osteoporosis in RA mice, but these effects could be effectively alleviated by R406; however, while *Pe-*derived EVs also induced an immune response, their ability to promote RA was relatively limited.

## Discussion

The EV characterization results indicated that the vesicles isolated in the present study were bacterial outer membrane vesicles (OMVs). However, we cannot rule out the presence of other forms of bacterial vesicles in the extracted samples.^24^ Therefore, we uniformly refer to the extracted vesicles as EVs in this study.^25^ EVs are enveloped by a phospholipid bilayer, which makes them less susceptible to host protease enzymes or antibiotics and therefore more structurally stable.^26^ Oral bacteria-derived EVs can be transported to the brain,^14,27,28^ liver,^29^ bones,^30^ or adipose tissue,^31^ with blood-mediated transport being a well-documented pathway as revealed by numerous studies. For periodontal pathogen-derived EVs, the likelihood of entering the bloodstream significantly increases following scaling or surgical procedures, as well as due to bacterial disruption of endothelial cell junctions.^32^ To simulate blood transport, we administered EVs via intraperitoneal injection to each mouse, following the methodology of previous studies.^29–31,33^ This modeling approach overcomes the challenges of EVs from oral gavage having difficulty entering the periodontal pocket and the inaccuracy in controlling the quantity of EVs entering the peripheral blood circulation.^28^ Intraperitoneal administration also circumvents the issue of non-*Pg* EV- or non-*Pe* EV-specific infections caused by periodontal ligation modeling. The subsequent model of collagen-induced arthritis (CIA) in mice via tail vein injection resulted in tail ulceration, so we also did not use this delivery method. The in vivo tracing results presented here demonstrated that *Pg* EVs were transported to the bone marrow and paws more quickly than were *Pe* EVs, indicating the potential for direct contact with osteoclasts. The main symptom of RA is the destruction of joint bone and cartilage caused by excessive osteoclastogenesis. Surprisingly, *Pg* EVs could induce osteoclastogenesis irrespective of RANKL-RANK signaling activation, resulting in strong osteoclastogenic activity. In contrast, *Pe*-derived EV-induced generation of multinucleated osteoclasts is rare, suggesting that *Pe*-derived EVs lack some key virulence factors, especially some components that promote late-stage fusion of osteoclasts.

Next, we explored which active components of EVs induce osteoclastogenesis. We found that the integrity of the EV structure is critical for the induction of osteoclastogenesis by *Pg* EVs. This may be due to the phospholipid membrane structure of EVs, which increases the internalization efficiency of virulence factors through host cell phagocytosis.^34,35^ Additionally, LPS, proteins, lipoproteins, and serine proteases clearly play a role in *Pg* EV- and *Pe* EV-induced osteoclastogenesis. LPS,^36^ fimbriae^37^ and lipoproteins^38,39^ serve as ligands for Toll-like receptor 2 (TLR2). The TLR2/MyD88/NF-κB signaling pathway can crosstalk with the Syk pathway, which may represent one of the mechanisms by which *Pg* EVs promote osteoclastogenesis.^36,40^ The study indicates that deficiency of TLR2 can suppress *Pg* LPS-induced osteoclastogenesis.^36^ *Pg* fimbriae induce osteoclastogenesis in RAW264 cells via TLR2,^19^ while Pro K effectively degrades *Pg* fimbriae.^41^ This suggests that Pro K inhibits osteoclastogenesis by degrading ligand proteins, including fimbriae, on the surface of EVs. In addition, degradation of lipoproteins using lipase can inhibit *Pg*-mediated inflammation^42^ and *Pg* EV-induced osteoclastogenesis.^38^ However, compared with *Pe* EVs, *Pg* EVs may encapsulate certain unique virulence factors, such as gingipains, which is reflected in the strong protein expression observed in the 40–55 kDa range during *Pg* EV characterization.^43^ Our previous studies confirmed the presence of gingipains in *Pg* EVs.^44^ Gingipains may have a synergistic effect in promoting osteoclastogenesis, which could also be a potential reason why *Pg*-derived EVs exhibit stronger osteoclastogenic induction capability than *Pe*-derived EVs.^45^ However, no treatment can completely block the osteoclastogenic activity induced by EVs, indicating that the osteoclastogenic activity of EVs depends on multiple factors and may involve synergistic effects. This also reflects the strong biological activity of EVs; if EVs act through a single virulence factor, they would be very susceptible to being countered and inactivated by host cells.

It has been proposed that some cytokines can promote osteoclastogenesis.^46,47^ We utilized neutralizing antibodies against TNF-α, IL-1β and IL-6, which attenuated the formation of osteoclasts induced by *Pg* EVs but had little influence on the effects of *Pe* EVs. This seems to coincide with the previously widespread belief that virulent *Pg* bacteria or *Pg* factors induce osteoclastogenesis by promoting TNF-α, IL-1β or IL-6 expression.^45,48^ However, our subsequent experiments demonstrated that if the culture medium contained TNF-α, IL-1β or IL-6 but not EVs, osteoclastogenesis could not be independently induced. These findings indicate that *Pg* EV-induced osteoclast generation is governed by noncytokine signaling pathways. Cytokines help amplify the ability of *Pg* EVs to promote osteoclastogenesis.

We subsequently performed RNA-seq analysis and identified the Syk gene as significantly upregulated. Syk, a 72 kD nonreceptor tyrosine kinase, is involved mainly in signaling cascades associated with immunity.^49^ The activation of Syk requires the engagement of classical immunoreceptors on the osteoclast membrane surface, such as T- and B-cell receptors (TCRs and BCRs, respectively) and Fc receptors.^50^ *Pg* EVs carry bioactive components that could potentially activate the aforementioned receptors and induce osteoclastogenesis independent of RANKL–RANK signaling, which warrants further investigation in the future. Syk activates the phospholipase Cγ2 (PLCγ2) enzyme and inositol 1,4,5-triphosphate (IP3) signaling cascade, leading to fluctuations in intracellular Ca^2+^ signals and thereby activating the transcription factor NFATc1, which is the most critical regulator of osteoclastogenesis.^51,52^ R406 is a highly selective Syk inhibitor that can suppress Syk and p-Syk protein expression.^53,54^ The prodrug of R406, fostamatinib, has been used for the treatment of chronic immune thrombocytopenia and RA.^55,56^ Here, R406 significantly inhibited osteoclast generation in the EV groups, and the inhibition rate was close to 100%. Although the RNA-seq results revealed no change in the Syk gene during RANKL-induced osteoclastogenesis, the inhibition of Syk expression led to impaired NFATc1 activation, resulting in the suppression of RANKL-induced osteoclast formation.^57^ Therefore, although Syk is specifically upregulated during EV-induced osteoclastogenesis, it also concurrently affects RANKL-induced osteoclastogenesis. In brief, we innovatively propose that *Pg*-derived EVs promote osteoclastogenesis by activating Syk. These findings provide a new research direction for investigating the mechanism of bone destruction mediated by *Pg*.

We further constructed a CIA mouse model. Recently, a study identifying damaging monoallelic Syk variants in patients with multiorgan inflammatory diseases demonstrated the core role of Syk phosphorylation-induced osteoclastogenesis in RA.^58^ This finding is consistent with our results showing that the Syk inhibitor R406 significantly attenuated *Pg* EV-promoted RA osteoclastogenesis and bone destruction. Additionally, as an upstream regulator of NF-κB, Syk plays a crucial proinflammatory role in various diseases.^59^ Therefore, inhibiting Syk further weakened the expression of TNF-α, IL-1β, and IL-6 mediated by *Pg* and *Pe* EVs, promoting the protection of RA joints and bones. These findings suggest that Syk could be a crucial hub in the response to infection by various pathogenic microorganisms in individuals with RA. However, the downstream pathways of Syk need further exploration. In RA, the secretion of RANKL by synovial cells serves as the primary inducer of osteoclastogenesis.^60,61^ T cells and B cells can secrete inflammatory cytokines such as TNF-α, IL-1β, or IL-6, which can promote RANKL expression in synovial fibroblasts.^61–63^ The possibility that *Pg* EVs indirectly affect osteoclastogenesis by acting on synovial cells, T cells, or B cells cannot be ruled out in in vivo experiments. However, in this study, we aimed to explore whether *Pg* EVs could further promote osteoclastogenesis through direct action, beyond the mechanisms mentioned above, thereby exacerbating RA bone destruction. In the future, investigating the effects of culture supernatants from *Pg* EVs-pretreated synovial cells, T cells, or B cells on osteoclastogenesis may provide a more comprehensive understanding of the indirect regulatory role of *Pg* EVs on osteoclasts in RA.

Although both *Pg* and *Pe* belong to the *Porphyromonas* genus and can promote the expression of inflammatory factors in mice, *Pe-*derived EVs have limited effects on RA, whereas *Pg-*derived EVs can significantly aggravate RA. These findings indicate that *Pe-*derived EVs may differ significantly from *Pg-*derived EVs in terms of the type and number of virulence factors; thus, *Pe-*derived EVs have a weak ability to promote osteoclastogenesis and bone destruction in RA. Compared with that of *Pe* EVs, the exacerbation of RA bone destruction by *Pg* EVs is species-specific rather than a universal immune response to bacterial infection. Furthermore, *Pg* EVs can disrupt endothelial intercellular junctions and increase vascular permeability through the hydrolytic action of gingipains,^11,64,65^ which may serve as a critical factor explaining why *Pg* EVs exhibit faster blood transport in mice compared to *Pe* EVs. The higher transport efficiency enables *Pg* EVs to enter and accumulate in the bone marrow cavity and paws earlier, thereby more effectively activating osteoclasts, resulting in a more potent exacerbation of RA-induced bone destruction compared to *Pe* EVs.

This study also has limitations. To elucidate the independence of Syk from RANKL signaling, the overexpression of downstream factors such as PLC alongside Syk knockdown would more clearly reveal this mechanism. Future inclusion of an independent control group for EVs or establishment of a periodontal ligation model would enable the comprehensive evaluation of the impact of *Pg* or PD on RA. In addition, investigating the impact of *Pg* EVs in the RA microenvironment on osteoclastogenesis through the activation of immune cells such as T cells and B cells would provide a more comprehensive understanding of the immunomodulatory role of *Pg* EVs. Future studies focusing on female CIA mice would contribute to elucidating the role of sex differences in *Pg* EV-induced osteoclastogenesis in RA.

**Fig. 13:**
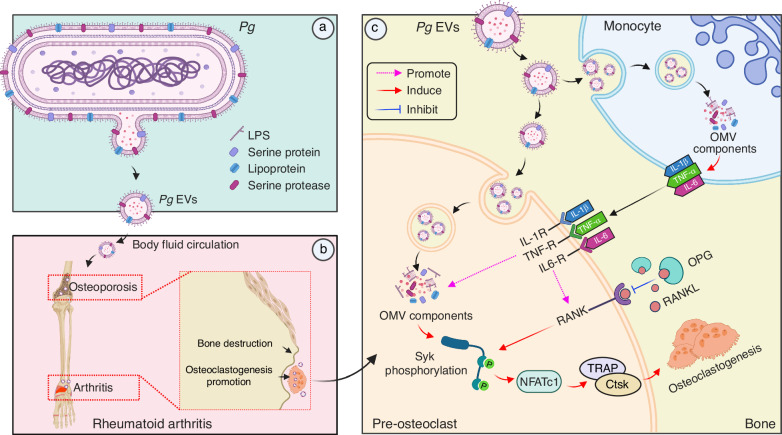
*Pg* EVs aggravate rheumatoid arthritis by promoting Syk-dependent osteoclastogenesis. **a** *Pg* can secrete nanometer-sized extracellular vesicles (EVs) through outer membrane budding. **b** *Pg* EVs were transported to the long bone and metacarpal joints of mice and came into direct contact with preosteoclasts. *Pg* EVs further exacerbated RA-induced joint bone destruction and osteoporosis by promoting osteoclastogenesis. **c** Mechanistically, *Pg* EVs induce osteoclastogenesis by activating Syk, and many active components of *Pg* EVs, such as LPS, proteins, lipoproteins, and proteases, might participate in this process. Moreover, TNF-α, IL-1β and IL-6 enhanced the ability of *Pg*-derived EVs to promote osteoclast generation

In summary, we demonstrated that *Pg* and *Pe* EVs can be rapidly transported to the bone marrow and paws of mice and contact monocytes or preosteoclasts directly. Owing to the synergistic initiation effect of multiple EV components (proteins, lipoproteins, and proteases) and the additional effect of cytokines (TNF-α, IL-1β and IL-6), *Pg*-derived EVs have a powerful ability to induce osteoclastogenesis. Furthermore, *Pg-*derived EVs can aggravate joint and bone destruction in RA mice by activating Syk-dependent osteoclastogenesis (Fig. 13a‒c). We propose a new mechanism by which the PD pathogen *Pg* may affect bone destruction in RA through circulating EVs. This study deepens our understanding of the significant pathogenic role of EVs from oral bacterial in RA and provides essential insights and strategies for the clinical treatment of RA patients with periodontal disease by targeting Syk.

## Materials and methods

### EV isolation and identification

*Pg* (ATCC33277) and *Pe* (ATCC35406) were cultured anaerobically in brain heart infusion (BHI) (Solarbio, Beijing, China; cat#: LA0360) supplemented with 0.5% yeast extract (Thermo Fisher Scientific, MA, USA; cat#: LP0021B), 10 μg/mL hemin (RYON, Shanghai, China; cat#: 16009-13-5) and 1 μg/mL vitamin K1 (MoFoKangYaoYe, Neijiang, China) at 37 °C for 4 days. The morphology and structure of intact bacteria were observed by SEM (SU-3500, HITACHI, Tokyo, Japan) and TEM (H-7650, HITACHI), respectively, as previously described.^29^ EVs were isolated according to a protocol we previously described.^29^ The protein concentration of the EVs was measured with a Bradford assay kit (Beyotime, Jiangsu, China; cat#: P0006C). Bacterial lysates and EVs (2 μg) were separated on 10% gels by SDS‒polyacrylamide gel electrophoresis (SDS‒PAGE) and then stained following the instructions of the Fast Silver Stain Kit (Beyotime; cat#: P0017S). EV morphology was observed by TEM as previously described.^29^ The diameter of the EVs was measured using a Zetasizer nano ZSP (Malvern Panalytical, England).

### Cell culture

Murine RAW264.7 monocytes were obtained from Procell Life Science & Technology Co., Ltd (cat#: CL-0190) and cultured in DMEM (Procell; cat#: PM150210) supplemented with 10% FBS (Procell; cat#: 164210-50) and 1% penicillin‒streptomycin solution (HyClone, UT, USA; cat#: SV30010). Cells that had been passaged fewer than fifteen times were used to induce osteoclast differentiation. RAW264.7 cells (2 × 10^3^ per well) cultured in 96-well plates were treated with sRANKL (75 ng/mL, PeproTech, NJ, USA; cat#: 315-11-10) or EVs (1 μg/mL) for 6 days. The medium containing sRANKL or EVs was changed every 2 days. After culture, the cells were subjected to tartrate-resistant acid phosphatase (TRAP) or F-actin staining.

### Intracellular fluorescence-based localization of EVs

EVs (1 μg/μL) were stained with DIL dye (10 μmol/L, Beyotime; cat#: C1036) at 37 °C for 30 min. The unincorporated dye was removed from the labeled EVs by repeated treatment with Total Exosome Isolation Reagent (3 times). To localize EVs in cells, RAW264.7 cells were stained with DIL dye-labeled EVs at 37 °C for 4 h. After washing with PBS, the cells were stained with Actin-Tracker Green-488 (1% v/v, Beyotime; cat#: C2201S) at 37 °C for 30 min. Then, the cells were washed with PBS again and stained with DAPI (1:1 000, Solarbio; cat#: C0060) at room temperature (RT) for 5 min. After washing with PBS, cells were imaged under a fluorescence microscope.

### TRAP staining

Cells were fixed with 5% formaldehyde for 10 min. The slides were deparaffinized and rehydrated through a graded ethanol series in distilled water. Then, the cells or slides were treated with 0.2% Triton X-100 in PBS for 5 min at RT. The cells or slides were subsequently stained with TRAP solution containing naphthol AS-MX phosphate (0.1 mg/mL, Sigma-Aldrich, MO, USA; cat#: N4875) and fast red TR salt (0.6 mg/mL, Sigma-Aldrich; cat#: F3381) in the presence of sodium acetate (50 mmol/L, pH = 5.0; Aladdin, Shanghai, China; cat#: S118649) and sodium tartrate (50 mmol/L, pH = 5.0; Aladdin; cat#: S304799) at 37 °C. TRAP-positive multinucleated cells containing three or more nuclei were counted as mature osteoclasts.

### Fibrous actin (F-actin) ring staining

Cells cultured in 96-well plates were fixed with 5% formaldehyde for 10 min and then rinsed twice with 0.1% Triton X-100 in PBS for 5 min each time at RT. The F-actin rings in the cells were subsequently stained with Actin-Tracker Red-Rhodamine (1% v/v, Beyotime; cat#: C2207S) dissolved in PBS supplemented with 0.1% Triton X-100 and 1% bovine serum albumin (BSA, Solarbio; cat#: A8020) for 45 min in the dark. After incubation, the cell nuclei were stained with DAPI (1:1 000, Solarbio) for 5 min and then washed twice with PBS. Finally, images were captured under a fluorescence microscope. All the images shown in this paper are merged images of actin (red fluorescence) and nuclei (blue fluorescence).

### siRNA transfection

RANK siRNA (siRANK) and negative control siRNAs (siNCs) were designed and synthesized by GenePharma (Shanghai, China). The targeting sequences for siRANK were 5′-GCGCAGACUUCACUCCAUAUU-3′ (sense) and 5′-UAUGGAGUGAAGUCUGCGCUU-3′ (antisense). Transfection was performed via INTERFERin^®^ (PolyPlus, Illkirch, France; cat#: 101000028). Briefly, for a 1 mL transfection system, 1 µg of siRANK or siNC was diluted in 100 µL of DMEM without FBS. After vortexing for 10 s, 1.5 µL of INTERFERin^®^ was added and incubated for 10 min at RT; then, the transfection mixture was added to 900 µL of fresh medium. For transfection, RAW264.7 cells were incubated in the above medium containing the transfection mixture for 6 h, and then the medium was changed to fresh medium containing RANKL or EVs and cultured for the indicated times.

### Real-time PCR

Total RNA was extracted from cells via TRIzol reagent (Takara, Shiga, Japan; cat#: 9109) according to the manufacturer’s protocol. cDNA was synthesized via a PrimeScript reverse transcription kit (Takara; cat#: RR036A). The expression of osteoclastic differentiation marker genes was determined via an ABI 7500 system (Applied Biosystems, CA, USA) with qPCR SYBR Premix Ex Taq™ (Takara; cat#: RR820A). Relative mRNA levels were calculated via the 2^(−ΔΔCt)^ method, with β-actin expression used as the normalization control. The sequences of the primers used are listed in Table S1.

### ELISA

Cytokine concentrations in supernatants or mouse serum were measured via ELISA kits from Thermo Fisher Scientific according to the manufacturer’s instructions. The following cytokines were assayed: mouse TNF-α (cat#: 88-7324-22), mouse IL-1β (cat#: 88-7013-22) and mouse IL-6 (cat#: 88-7064-22).

### RNA-seq analysis

Total RNA was extracted by using TRIzol (Takara). Technical support for RNA-sequencing analysis was provided by CapitalBio Technology (Beijing, China). The parameters for classifying differentially expressed genes (DEGs) were ≥2-fold differences (|log_2_FC| ≥ 1, FC: the fold change in expression) in transcript abundance and *P* ≤ 0.05. Kyoto Encyclopedia of Genes and Genomes (KEGG) analysis of the identified pathways enriched with DEGs was performed. Gene set enrichment analysis (GSEA) was performed with GSEA software (http://www.gsea-msigdb.org/). All the data except the GSEA data were visualized with Hiplot (https://hiplot.cn).

### Western blotting

Total protein was extracted using a cell lysate buffer (Beyotime; cat#: P0013) and then separated by SDS‒PAGE. The proteins were subsequently transferred onto polyvinylidene fluoride (PVDF) membranes (Merck, Darmstadt, Germany; cat#: IPVH00011). The membranes were blocked with QuickBlock™ Blocking Buffer (Beyotime; cat#: P0252) for 15‒20 min and then incubated with QuickBlock™ primary antibody dilution buffer (Beyotime; cat#: P0239) and diluted spleen tyrosine kinase (Syk, 1:1 000, Cell Signaling Technology, MA, USA; cat#: 13198), phospho-Syk (p-Syk, 1:500, ABclonal, Wuhan, China; cat#: AP0542), and *β*-actin (1:5 000, Proteintech, Wuhan, China; cat#: 66009-1-Ig) antibodies at 4 °C overnight. The membranes were washed with PBS-Tween and incubated with DyLight 680 (cat#: A23710) or 800 (cat#: A23920) secondary antibodies (1:10 000, Abbkine, Wuhan, China) for 1 h. The stained protein bands were visualized via the Odyssey^®^ DLx imaging system (LI-COR, NE, USA).

### Animal experiments

The animal studies were performed in accordance with the guidelines approved by the Institutional Animal Care Committee of China Medical University (CMU20231191) and the Guide for the Care and Use of Laboratory Animals published by the US National Institutes of Health and Regulations and the ARRIVE guidelines. 7-week-old male DBA1/JGpt mice were purchased from Gempharmatech Co., Ltd (Jiangsu, China; no. N000219). All the mice were kept in a standard environment with controlled temperature and humidity and a 12-h light/dark cycle under specific-pathogen-free conditions within randomly assigned microisolator cages (5 mice/cage) with food and water.

To track the transport of EVs in vivo, EVs (1 μg/μL) were incubated with DiR iodide (5 μmol/L; Yeasen, Shanghai, China; cat#: 40757ES25) for 30 min at 37 °C. 7-week-old male mice were randomly assigned to three groups (*n* = 3 per group): (1) the PBS group. (2) the *Pe* EV group. (3) the *Pg* EV group. Then, 50 μL of PBS or DiR dye-labeled EVs (1 μg/μL) were injected intraperitoneally into the mice. At 1 and 3 h postinjection, the mice were sacrificed, and DiR fluorescence was analyzed in various organs, including the heart, lung, liver, kidney, spleen, femur, tibia and paw, using an AniView100 Multimode Live Animal Imaging System (Boluteng Biological Technology Co., Ltd, Guangzhou, China).

To verify the effects of EVs on bone destruction and the abovementioned mechanisms in vivo, we constructed a CIA mouse model. To ensure the success rate of CIA modeling, we selected male DBA mice, which are more sensitive to CII immunity.^66^ Thirty mice were randomly assigned to six groups (*n* = 5 per group): (1) In the PBS/DMSO group (blank), nonimmunized mice received solvent-containing DMSO injections each time. (2) In the CII/PBS/DMSO group (RA), RA-immunized mice received solvent DMSO injections each time. (3) In the CII/*Pe* EVs/DMSO group (RA + *Pe* EVs), RA-immunized mice received *Pe* EVs and solvent DMSO injections each time. (4) In the CII/*Pe* EVs/R406 group (RA + *Pe* EVs +R406), RA-immunized mice received *Pe* EVs and R406 (dissolved in DMSO) injections each time. (5) In the CII/*Pg* EVs/DMSO group (RA + *Pg* EVs), RA-immunized mice received *Pg* EVs and solvent DMSO injections each time. (6) In the CII/*Pg* EVs/R406 group (RA + *Pg* EVs +R406), RA-immunized mice received *Pg* EVs and R406 injections each time. Briefly, 7-week-old male mice were immunized with bovine type II collagen (CII, 2 mg/mL; Chondrex, WA, USA; cat#: 7008) emulsified in an equal volume of complete Freund’s adjuvant (CFA; Chondrex; cat#: 20022). Then, 100 µL of the emulsion was injected subcutaneously at the base of the tail. An equal volume of PBS was injected into the negative control group without immunization. At 3 weeks after primary immunization, the mice were administered a booster immunization dose of CII (2 mg/mL) emulsified in an equal volume of incomplete Freund’s adjuvant (IFA, Chondrex; cat#: 7002). An equal volume of PBS was injected into the negative control group without immunization. At the same time as the second immunization, EVs were intraperitoneally injected with or without R406 (50 μg, Aladdin; cat#: R126996) every 3 days for 2 consecutive weeks. The non-R406-injected group was injected with the same volume of the solvent DMSO. All the samples were included in our analysis. The severity of arthritis was scored by a blinded examiner via a severity scoring system with scores ranging from 0 to 4 according to the instructions of Chondrex. Scoring was performed as follows: 0: no swelling or redness; 1: mild but definite redness and swelling of the ankle or wrist or apparent redness and swelling limited to individual digits, regardless of the number of affected digits; 2: moderate redness and swelling of the ankle or wrist; 3: severe redness and swelling of the entire paw, including the digits; and 4: maximally inflamed limb with involvement of multiple joints. At the indicated time points, the mice were euthanized, and blood was collected from the heart to obtain serum. The spleen, femur, tibia, and ankle joint, along with the entire paw, were dissected and fixed in a 4% paraformaldehyde solution.

### Micro-CT analysis

Micro-CT (SkyScan 1276, Bruker, MA, USA) scans were performed to evaluate the ankle joint along with the entire paw, with an operating voltage of 55 kV and an operating current of 200 μA at an isotropic voxel size of 20 μm. The femur was scanned with an operating voltage of 55 kV and an operating current of 72 μA at an isotropic voxel size of 4 μm. Three-dimensional reconstruction of the obtained images was performed with CTvox software. The bone volume (BV), tissue volume (TV) and trabecular number (Tb.N) were analyzed with CTAn software.

### Hematoxylin & eosin (H&E) staining

The femur, ankle and paw were subsequently demineralized with 10% EDTA for 4 weeks. The samples were subsequently embedded, dehydrated, and sectioned. For H&E staining, the sectioned samples were deparaffinized, hydrated and stained with hematoxylin for 10 min. Then, the sections were sequentially soaked in water 3 times, in 1% (v/v) hydrochloric acid alcohol for 10 min, in water 3 times, in eosin for 5 min, and in water for 3 min. Finally, the sections were dehydrated and sealed with neutral gum. Scoring of cartilage erosion in arthritic joints was evaluated according to the “SMASH” recommendations.^67^

### Immunohistochemistry

The sections were deparaffinized and incubated with pepsin (MXB Biotechnologies, Fuzhou, China; cat#: DIG-3009) at 37 °C for 30 min for antigen repair. Afterward, the sections were incubated with Syk (1:300, Cell Signaling Technology; cat#: 13198) or p-Syk primary antibodies (1:500, Abcam, Cambridge, England; cat#: ab300398) overnight at 4 °C and then stained using a DAB detection kit according to the manufacturer’s instructions (Gene Tech, Wuhan, China; cat#: GK600705). Counterstaining was performed with 0.1% hematoxylin (Servicebio, Wuhan, China; cat#: G1004).

### Statistical analysis

The data are presented as the means ± standard deviations (SDs). Dunnett’s test was applied for comparisons of multiple groups with one independent variable following one-way analysis of variance (ANOVA). Student’s *t* test was used for comparisons between two independent groups. All the analyses were performed with a significance level of *α* = 0.05.

## Supplementary information

Supplementary Table and Figures

## Data Availability

The data are available from the corresponding authors upon reasonable request.
