# Supplementary material for: Porphyromonas gingivalis-derived extracellular vesicles aggravate bone destruction in rheumatoid arthritis by promoting Syk-dependent osteoclastogenesis
Source: Int J Oral Sci. 2026 Feb 5;18:6. doi: 10.1038/s41368-025-00416-1 (PMC12873129; doi:10.1038/s41368-025-00416-1)
Supplement: Supplementary file 1 — Supplementary Table and Figures [file 41368_2025_416_MOESM1_ESM.docx]

Supplementary Information

***Porphyromonas gingivalis*****-derived extracellular vesicles** **aggravate bone destruction in** **rheumatoid arthritis by promoting Syk-dependent** **osteoclastogenesis**

Jiajie Guo^1^, Qiujing Qiu^1^, Xiaoyuan Yan^1^, Zeying Zhang^1^, Xiyue Zhang^1^, Na An^2^ Chengcheng Yin^3,4^, Di Yang^1^, Hirohiko Okamura^5^, Kaya Yoshida^6^, Hongchen Sun^7*^, Lihong Qiu^1*^

^1^Department of Endodontics, School and Hospital of Stomatology, China Medical University, Liaoning Provincial Key Laboratory of Oral Diseases, Shenyang, P. R. China.

^2^Department of Orthodontics, School and Hospital of Stomatology, China Medical University, Liaoning Provincial Key Laboratory of Oral Diseases, Shenyang, P. R. China.

^3^School and Hospital of Stomatology, Jilin University, Changchun, P. R. China.

^4^Jilin Provincial Key Laboratory of Tooth Development and Bone Remodeling, Changchun, P. R. China.

^5^Department of Oral Morphology, Graduate School of Medicine, Dentistry and Pharmaceutical Sciences, Okayama University, Okayama, Japan

^6^Department of Oral Healthcare Education, Institute of Biomedical Sciences, Tokushima University Graduate School, Tokushima, Japan

^7^Department of Oral Pathology, School and Hospital of Stomatology, China Medical University, Liaoning Provincial Key Laboratory of Oral Diseases, Shenyang, P. R. China.

**Table S1. Primer sequences**

| **Target** | **Forward** | | **Reverse** |
| --- | --- | --- | --- |
|  |  |  |  |
| **NFATc1** | | CCGTTGCTTCCAGAAAATAACA | TGTGGGATGTGAACTCGGAA |
| **OSCAR** | | TGCATGCCGTGCTGACTTC | AAGGTCACGTTGATCCCAGGAG |
| **Cathepsin K** | | CAGCAGAACGGAGGCATTGA | CTTTGCCGTGGCGTTATACATACA |
| **TRAP** | | GGGACAATTTCTACTTCACTGGAG | TCAGAGAACACGTCCTCAAAGG |
| **RANK** | | AGAAGACGGTGCTGGAGTCT | TAGGAGCAGTGAACCAGTCG |
| **TNF-α** | | CCCTCACACTCAGATCATCTTCT | GCTACGACGTGGGCTACAG |
| **IL-1β** | | GCAACTGTTCCTGAACTCAACT | ATCTTTTGGGGTCCGTCAACT |
| **IL-6** | | CACATGTTCTCTGGGAAATCGTGGA | TCTCTCTGAAGGACTCTGGCTTTGT |
| **β-actin** | | GGCTGTATTCCCCTCCATCG | CCAGTTGGTAACAATGCCATGT |

**
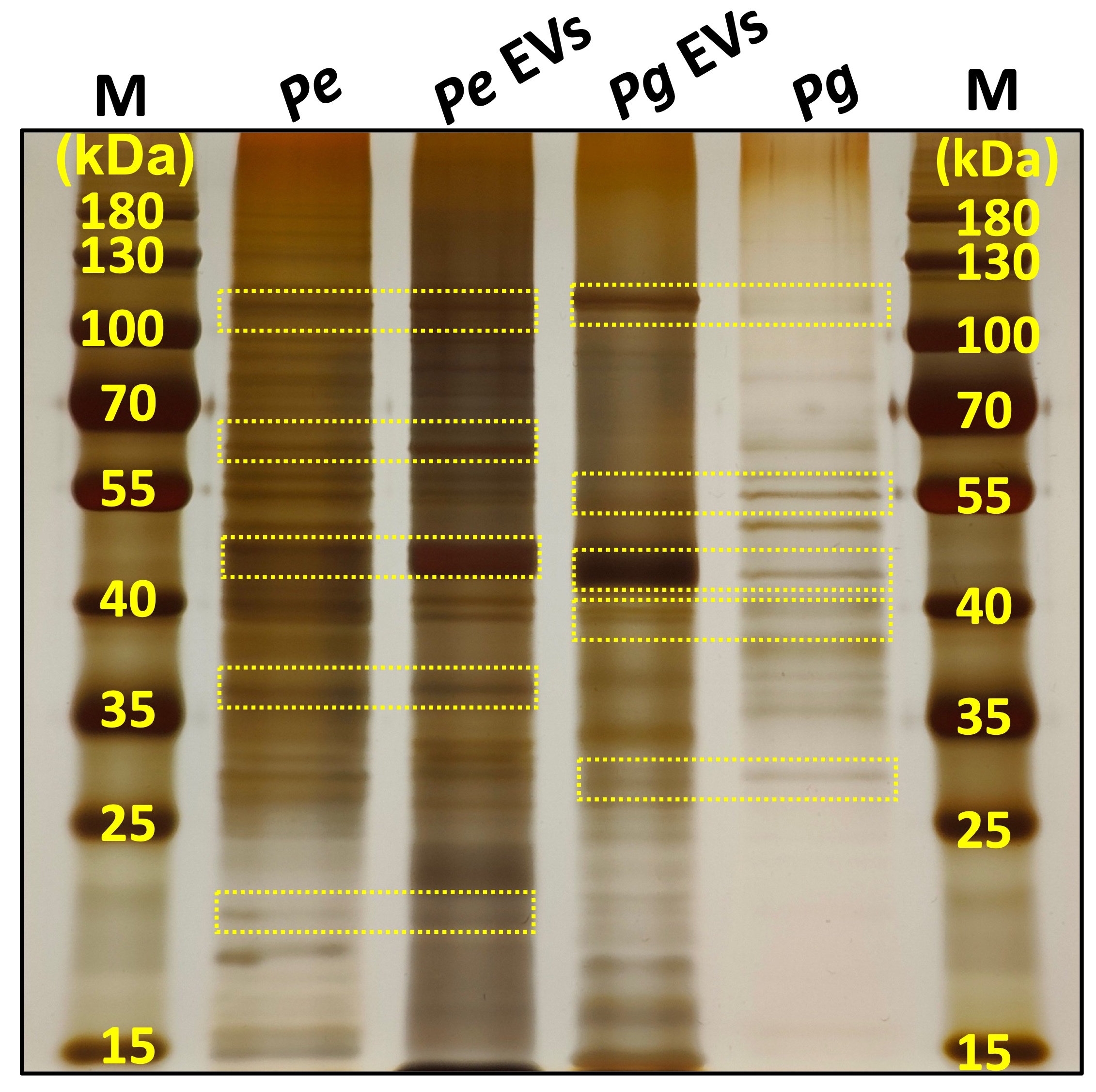
**

**Fig. S1** Silver staining of SDS‒PAGE gels showing the protein content of *Pe* and *Pg* EVs. The yellow box shows the proteins in EVs that were identical to those found in the parent bacteria.

**
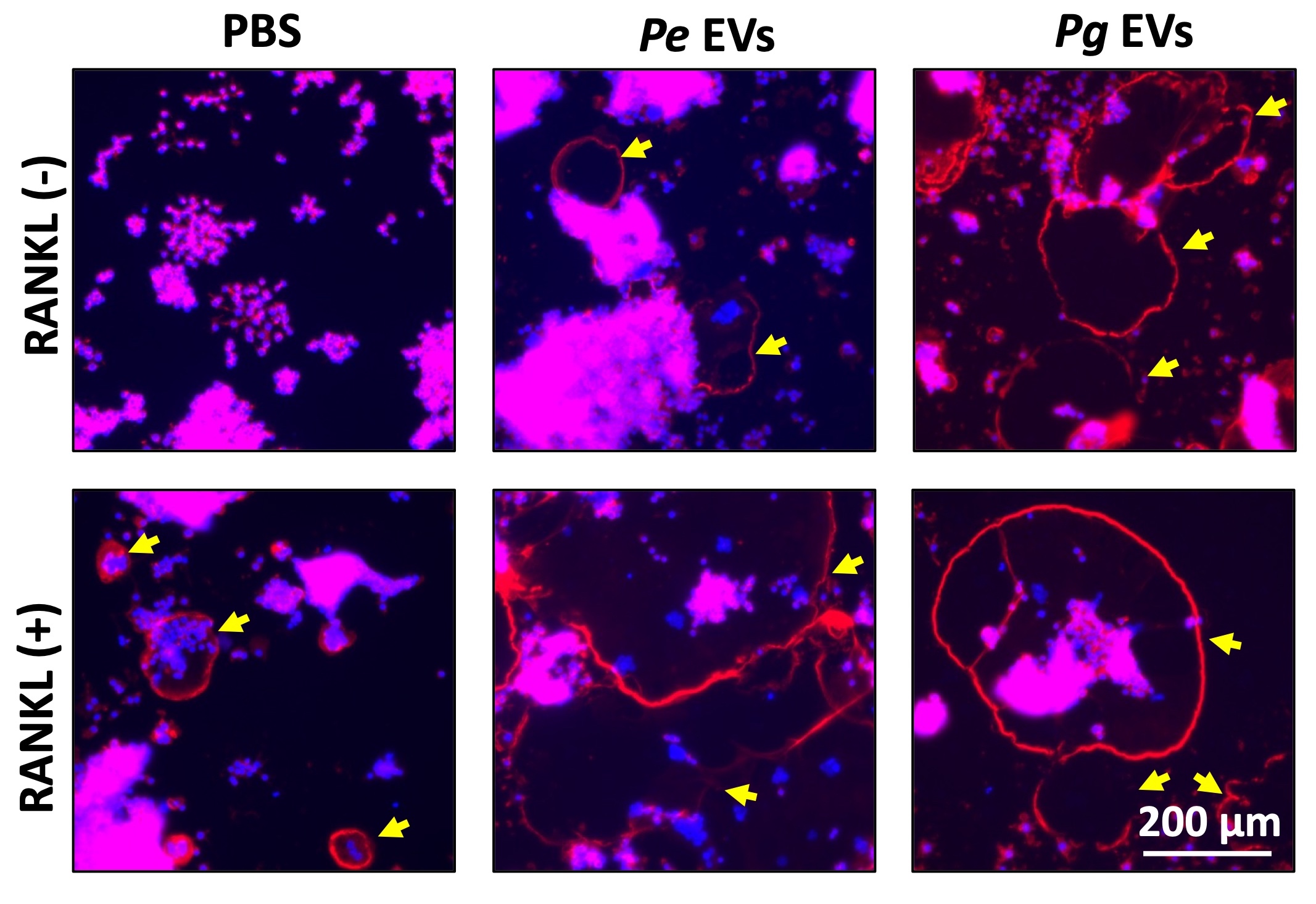
**

**Fig. S2** RAW264.7 cells were induced with EVs with or without RANKL for 6 days, and actin ring staining was performed.

**
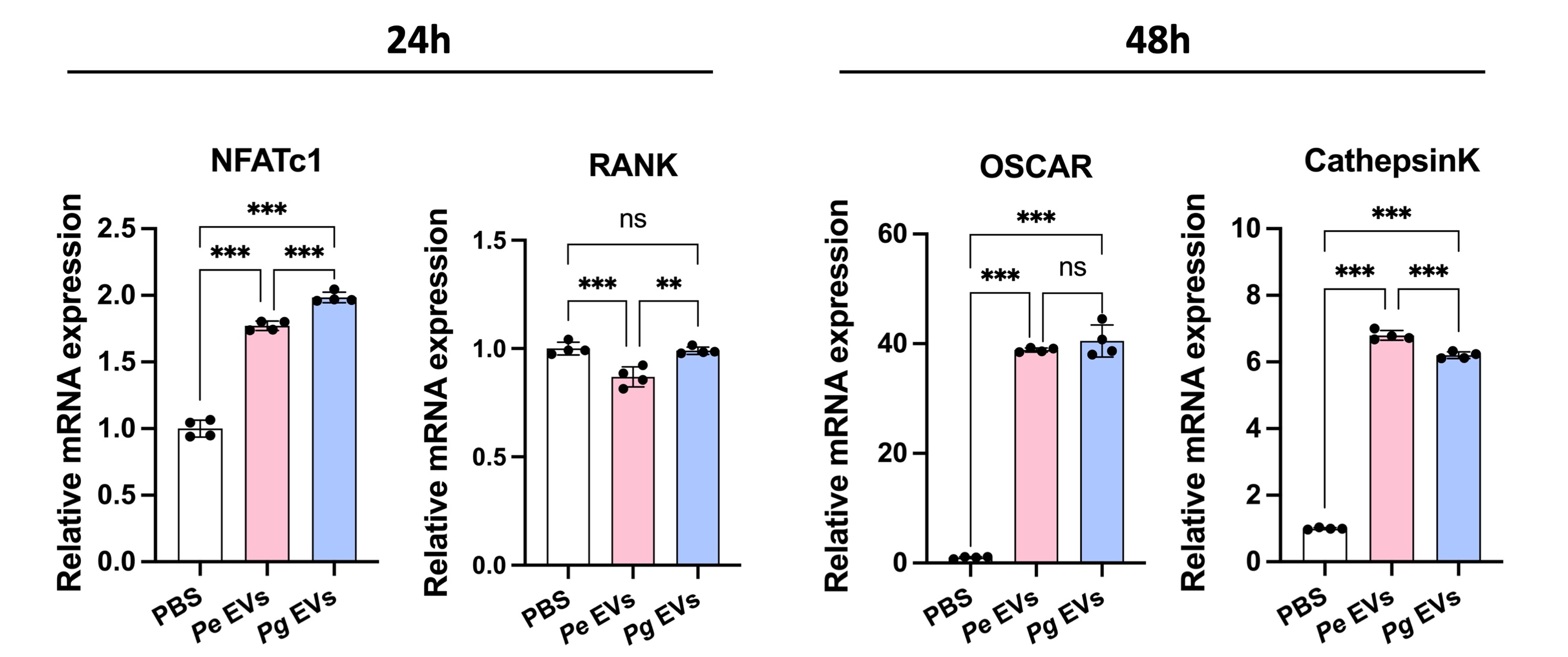
**

**Fig. S3** EVs promoted the expression of marker genes associated with osteoclast differentiation in vitro. RAW264.7 cells were treated with EVs for 24 or 48 h, and the expression of osteoclast generation marker genes was measured by RT‒PCR. ns *P* > 0.05, ** *P* < 0.01, *** *P* < 0.001


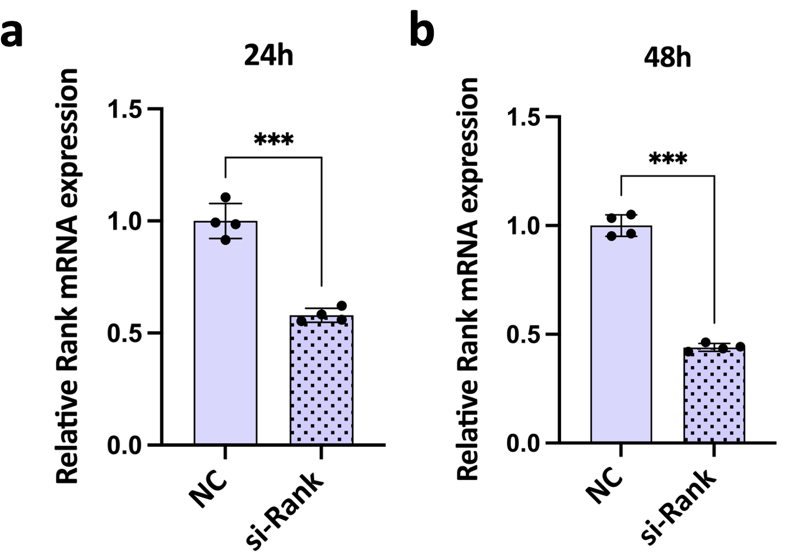


**Fig. S4** Inhibition efficiency of si-RANK. RAW264.7 cells were transfected with RANK si RNA for 6 h. Then, the medium containing the si RNA was changed to fresh medium. After 24 or 48 h, the RNA expression of RANK was confirmed by RT‒qPCR. *** *P*<0.001.

**
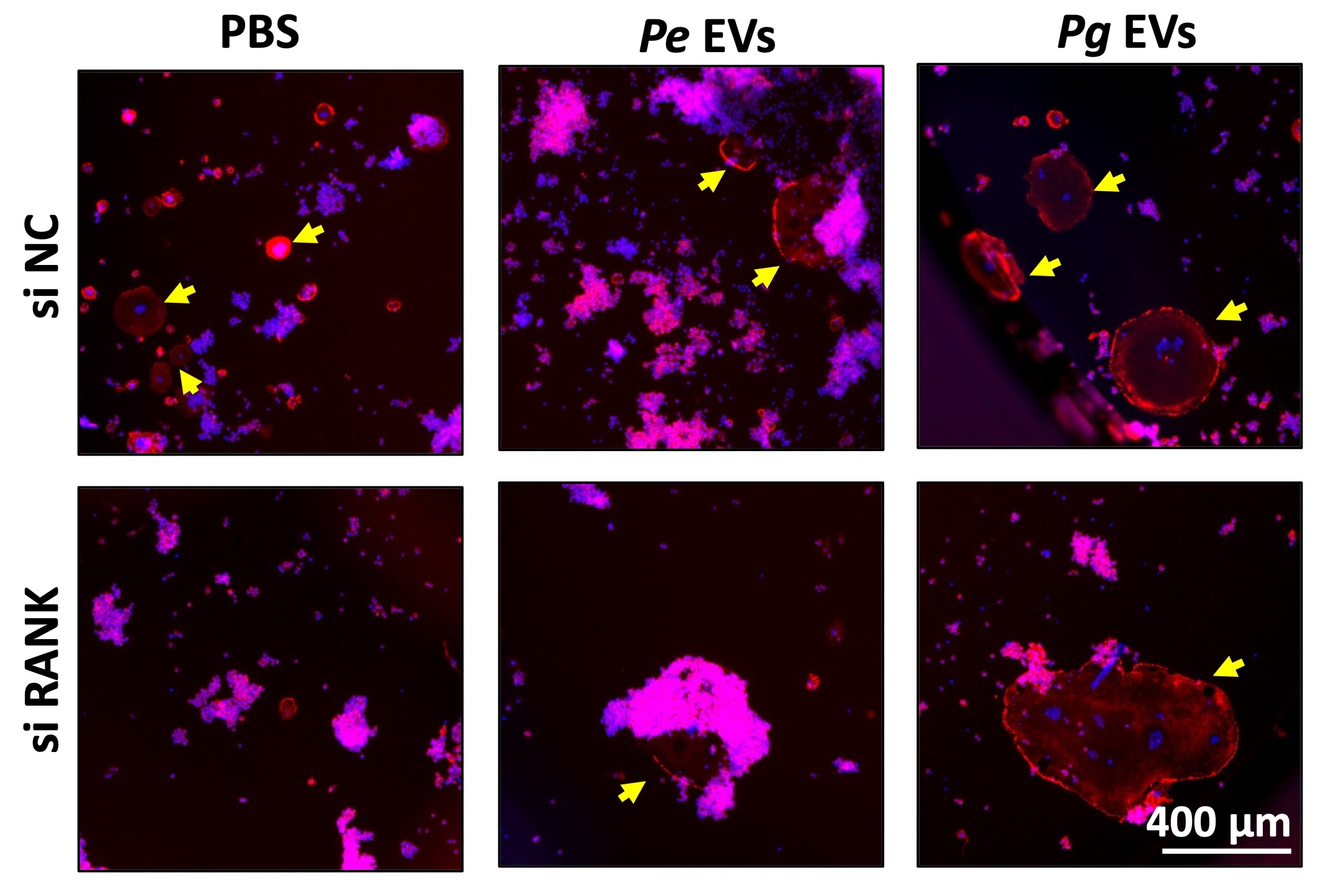
**

**Fig. S5** RAW264.7 cells were treated with EVs and RANKL for 6 days with or without RANK siRNA (siRANK), and actin ring staining was performed.


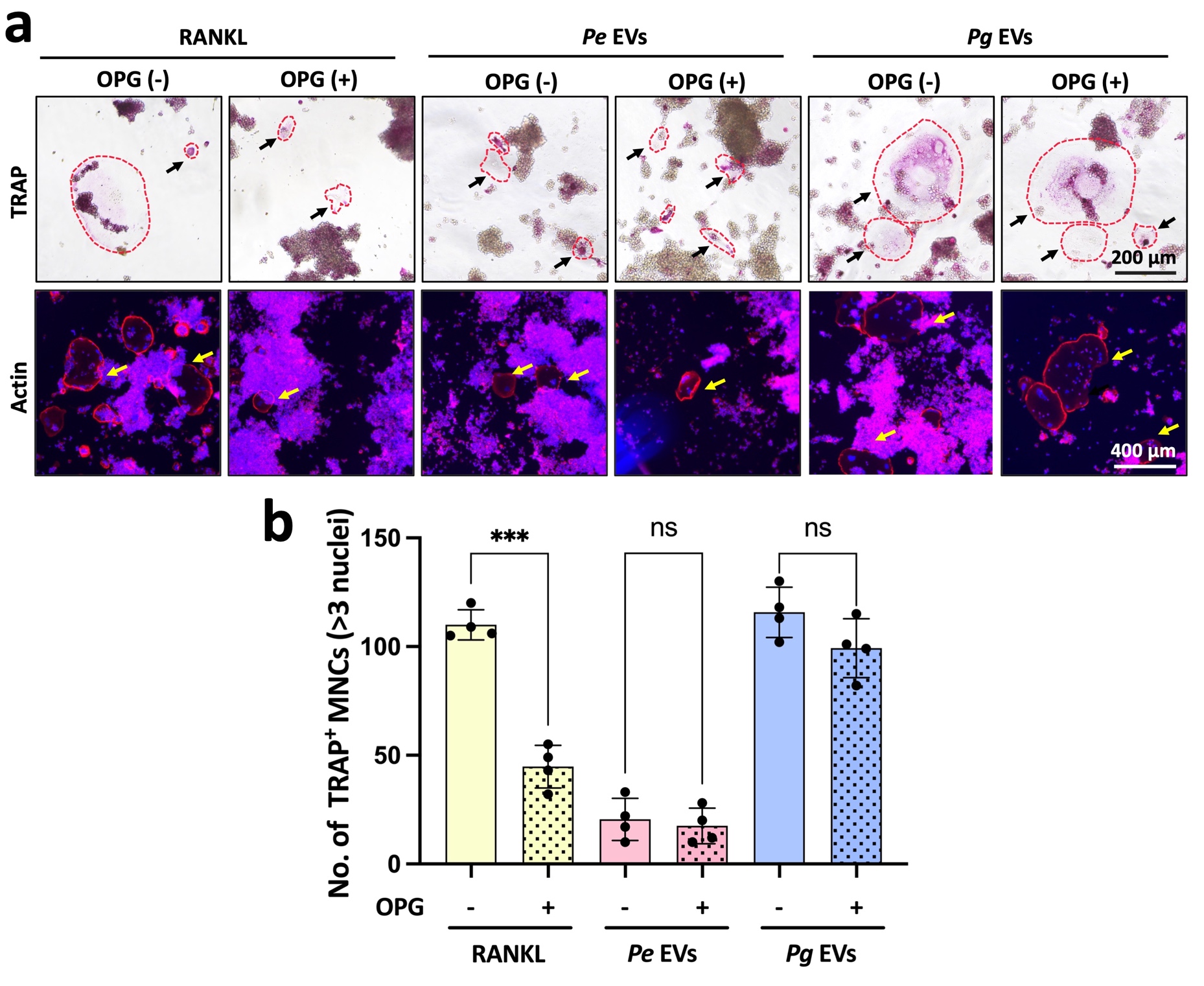


**Fig. S6** OPG inhibited RANKL- but not EV-induced osteoclastogenesis. Osteoprotegerin (OPG) is a decoy receptor for RANKL. **a** RAW264.7 cells were treated with RANKL and EVs in the presence or absence of OPG (75 ng/mL, Solarbio, Cat# P00873) for 6 days, after which TRAP staining and actin ring staining were performed. **b** TRAP-positive multinuclear cells with more than 3 nuclei were counted. The upper image shows the stained wells of each group. ns *P* > 0.05, *** *P* < 0.001.

**
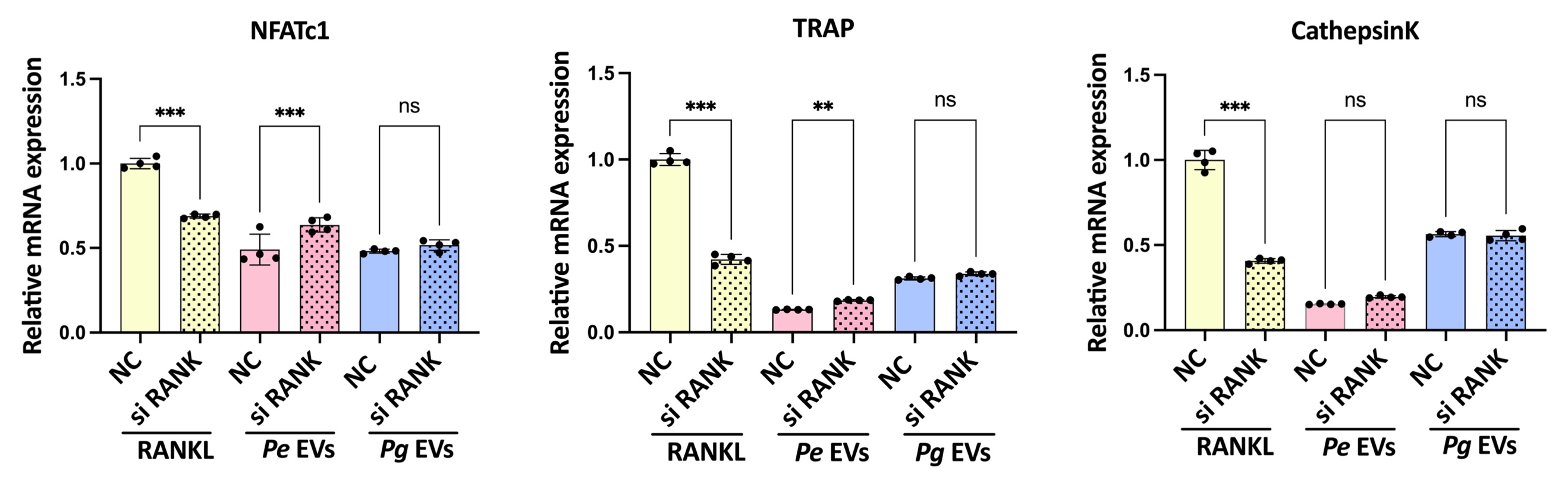
**

**Fig. S7** Knockdown of RANK in RAW264.7 cells does not affect the expression of osteoclast differentiation marker genes induced by EVs. RAW264.7 cells were treated with EVs and RANKL for 6 days with or without RANK siRNA (siRANK), and the relative expression of osteoclast generation marker genes was analyzed. ns *P* > 0.05, ** *P* < 0.01, *** *P* < 0.001.


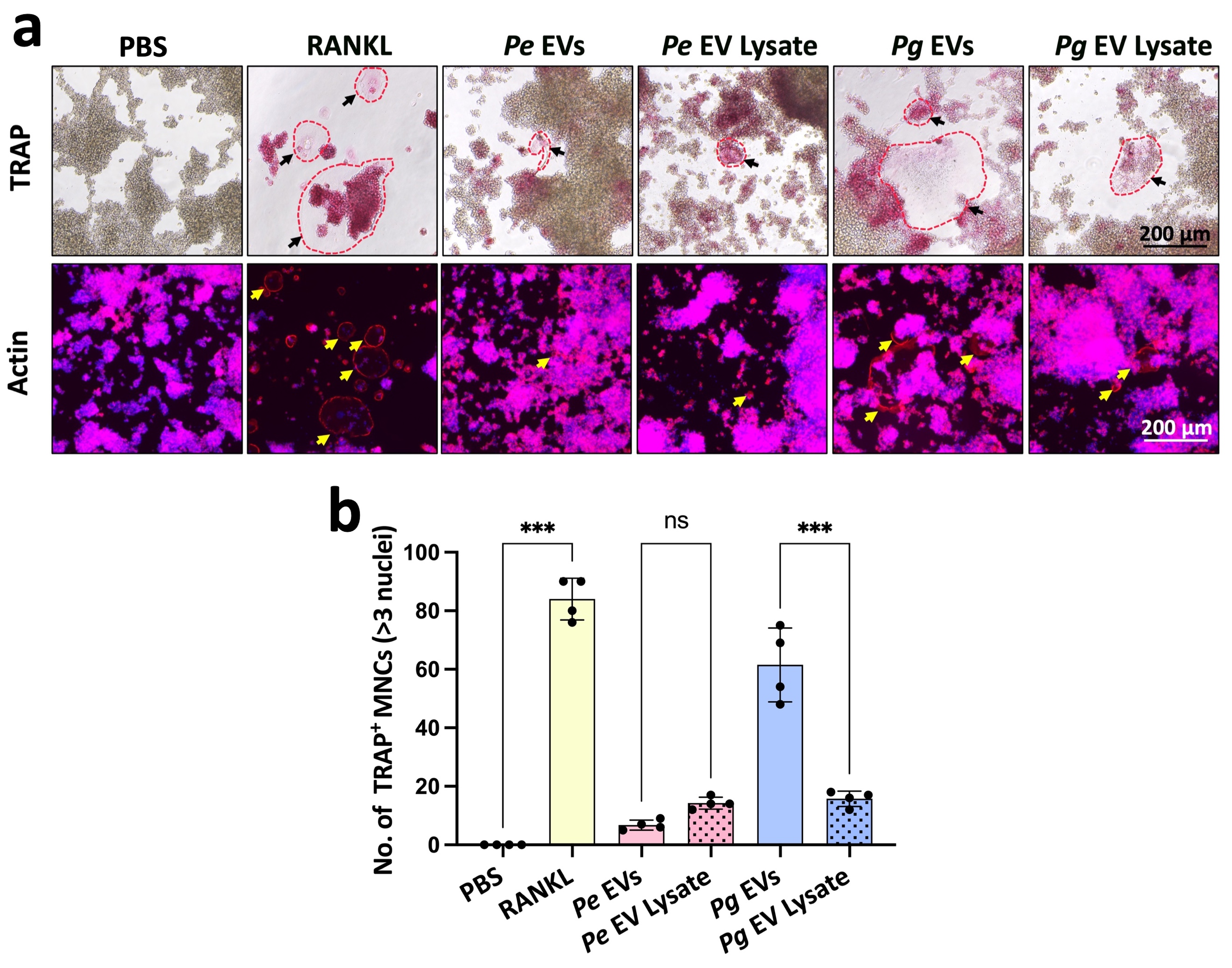


**Fig. S8** Vesicular structure disruption inhibits EVs-induced osteoclastogenesis. **a** EVs were frozen and thawed 5 times, followed by sonication for 30 s. Then, RAW264.7 cells were treated with EVs or EV lysate (1 μg/mL) for 6 days, and TRAP staining (upper plot) and actin ring staining (bottom plot) were performed. The red dashed line and arrows represent TRAP-positive osteoclasts. **b** TRAP-positive multinucleated cells with more than 3 nuclei were counted. ns *P* > 0.05, *** *P* < 0.001.

**
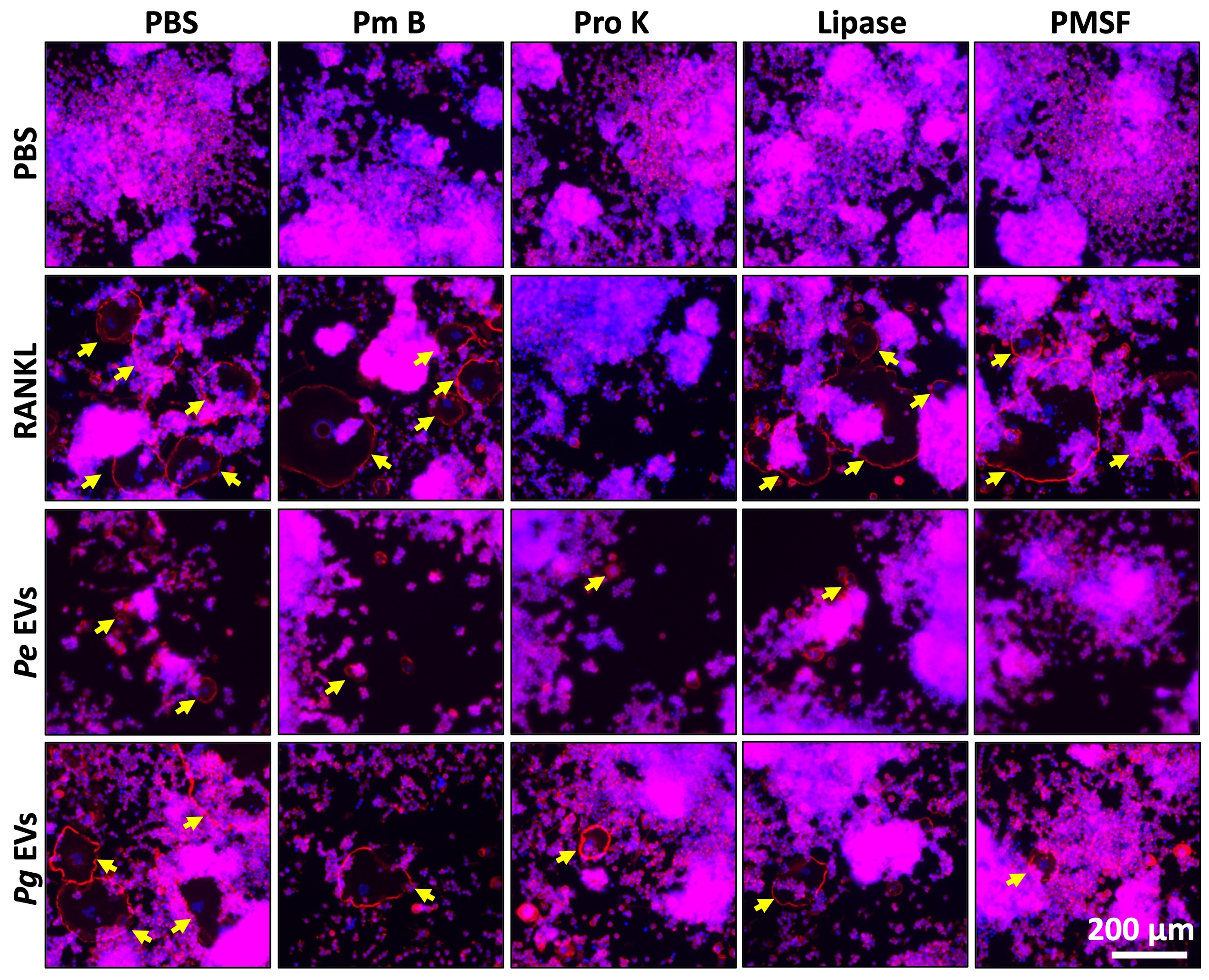
**

**Fig. S9** EVs or RANKL were pretreated with Pm B, Pro K, lipase and PMSF for 1 h at RT and then incubated at 4 °C. RAW264.7 cells were subsequently treated with Pm B-, Pro K-, lipase-, and PMSF-pretreated EVs or RANKL for 6 days, after which actin ring staining was performed.

**
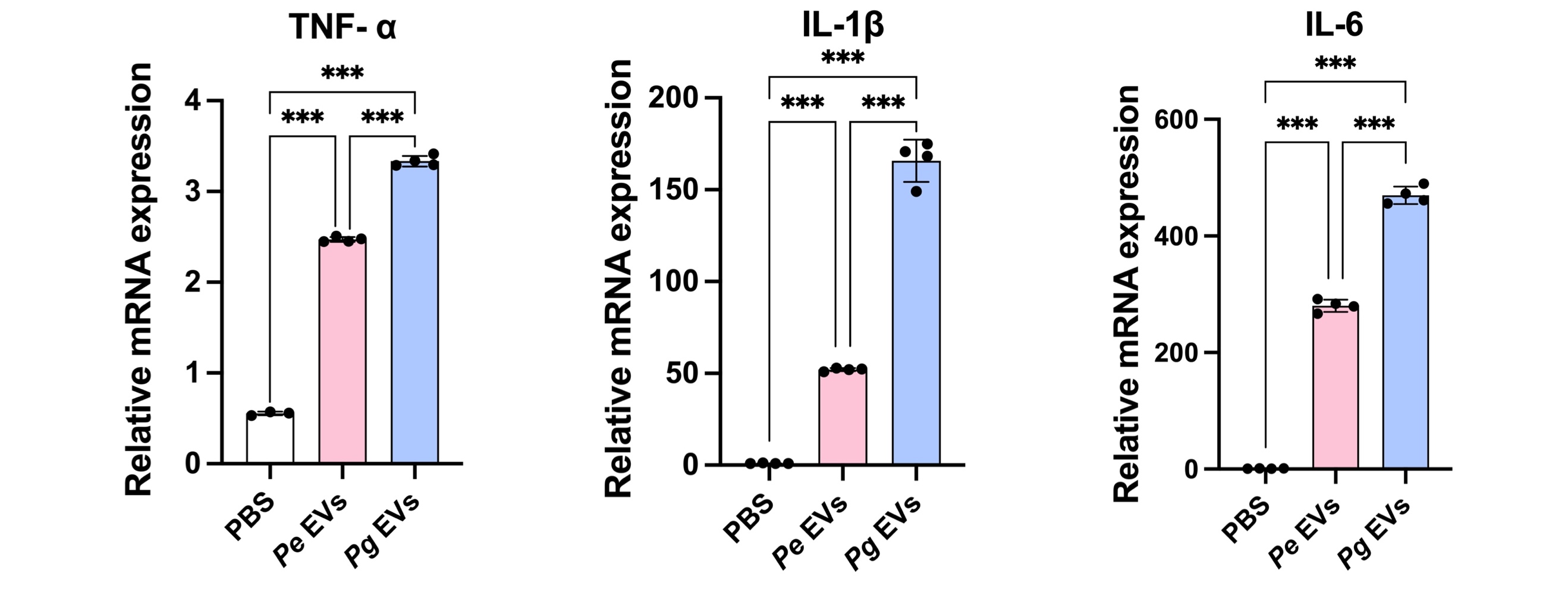
**

**Fig. S10** EVs promote cytokine transcription in RAW264.7 cells. RAW264.7 cells were treated with EVs for 48 h, and the relative gene expression of cytokines, including TNF-α, IL-1β, and IL-6 was analyzed. *** *P* < 0.001.

**
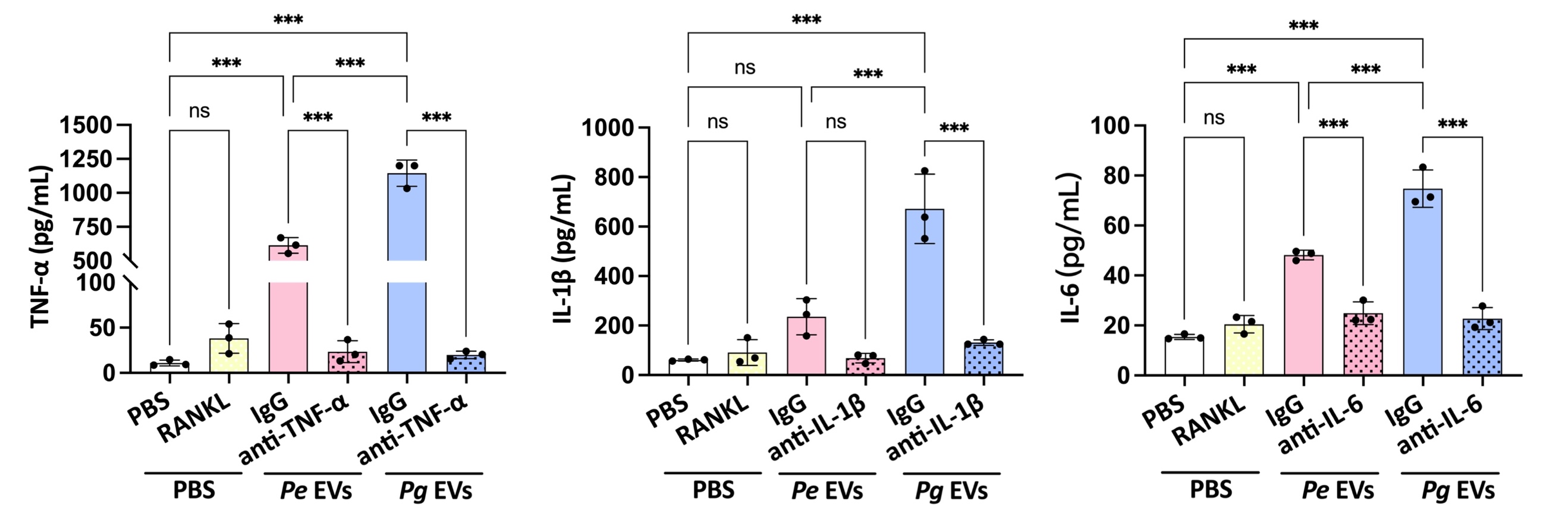
**

**Fig. S11** Neutralizing antibodies downregulate the levels of TNF-α, IL-1β, and IL-6 in the culture supernatant of RAW264.7 cells induced by EVs. RAW264.7 cells were treated with EVs in the presence of neutralizing antibodies against TNF-α, IL-1β, IL-6 or IgG for 6 days, and RANKL was used as a positive control. ELISA was performed to determine the concentrations of TNF-α, IL-1β and IL-6 in the supernatants of the cell cultures. ns *P* > 0.05, *** *P* < 0.001.

**
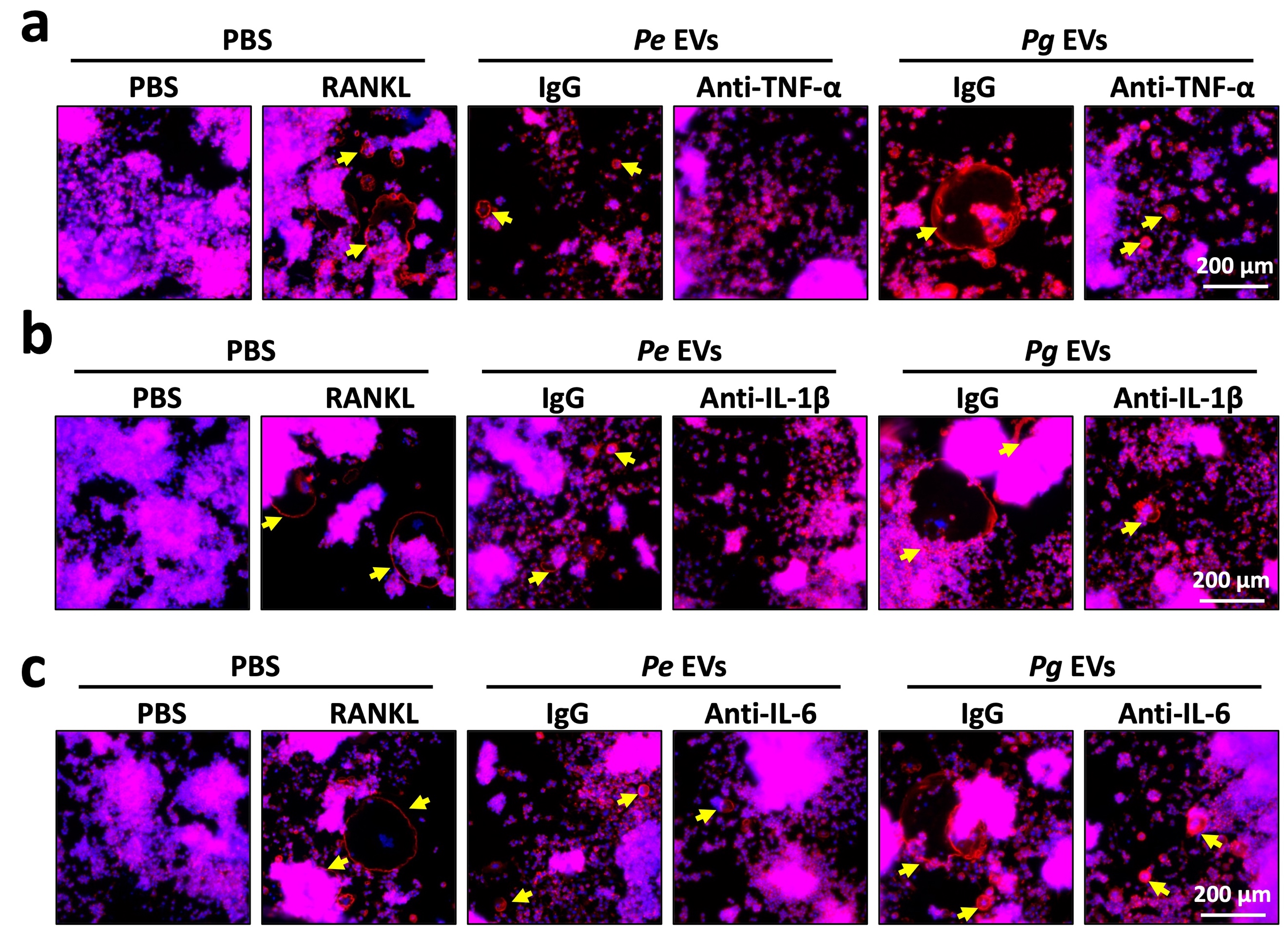
**

**Fig. S12** RAW264.7 cells were treated with EVs in the presence of neutralizing antibodies against TNF-α, IL-1β, IL-6 or IgG for 6 days, and RANKL was used as a positive control. Actin ring staining was performed.


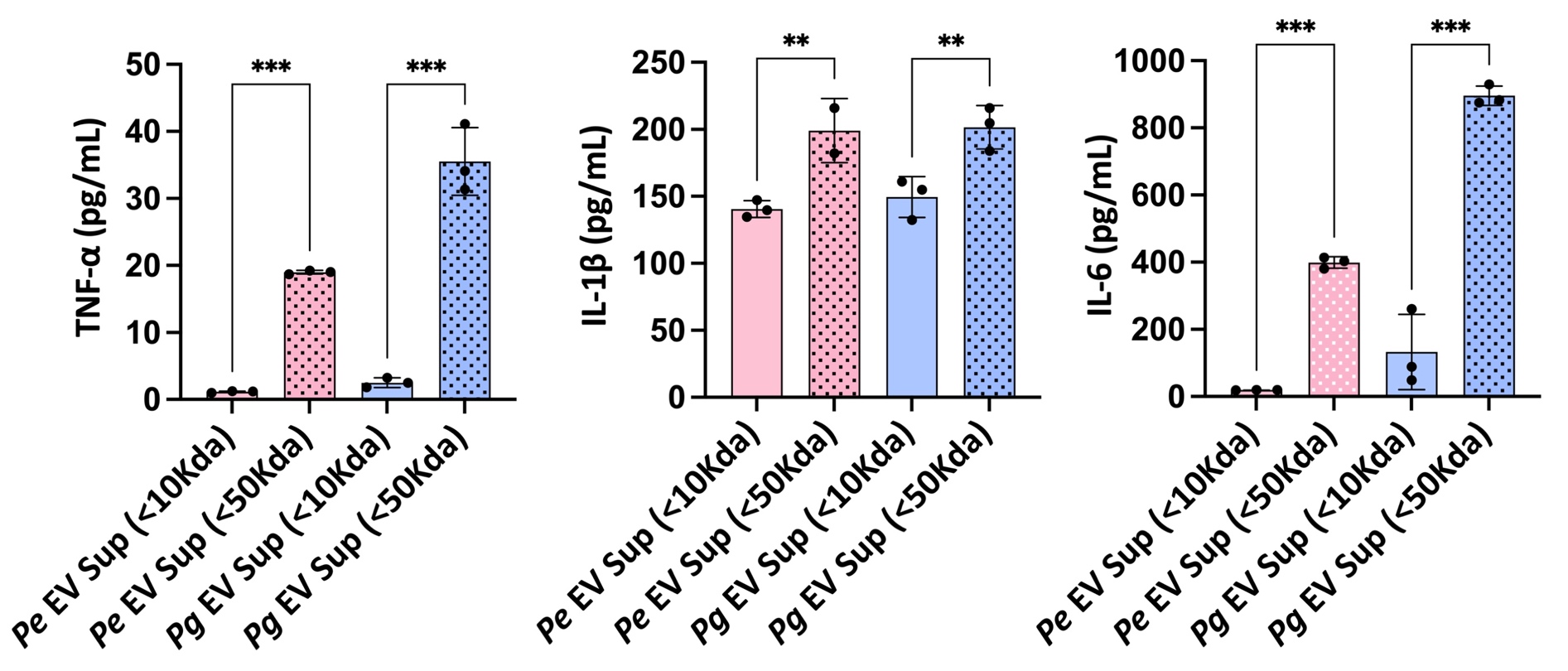


**Fig. S13** The levels of TNF-α, IL-1β, and IL-6 in culture supernatants prepared by different methods. RAW264.7 cells were treated with EVs for 2, 4, or 6 days, and the cell culture supernatant was collected at each time point. The supernatant was subsequently separated in ultrafiltration centrifuge tubes with different pore sizes to remove EVs, and supernatants containing TNF-α, IL-1β, and IL-6 (Sup < 50 kDa) or lacking TNF-α, IL-1β, and IL-6 (Sup < 10 kDa) were obtained. Cell culture supernatants collected at different time points (2, 4, and 6 days) were mixed, and ELISA was performed to measure the concentrations of TNF-α, IL-1β, and IL-6 in the mixed supernatants. ** *P* < 0.01, *** *P* < 0.001.

**
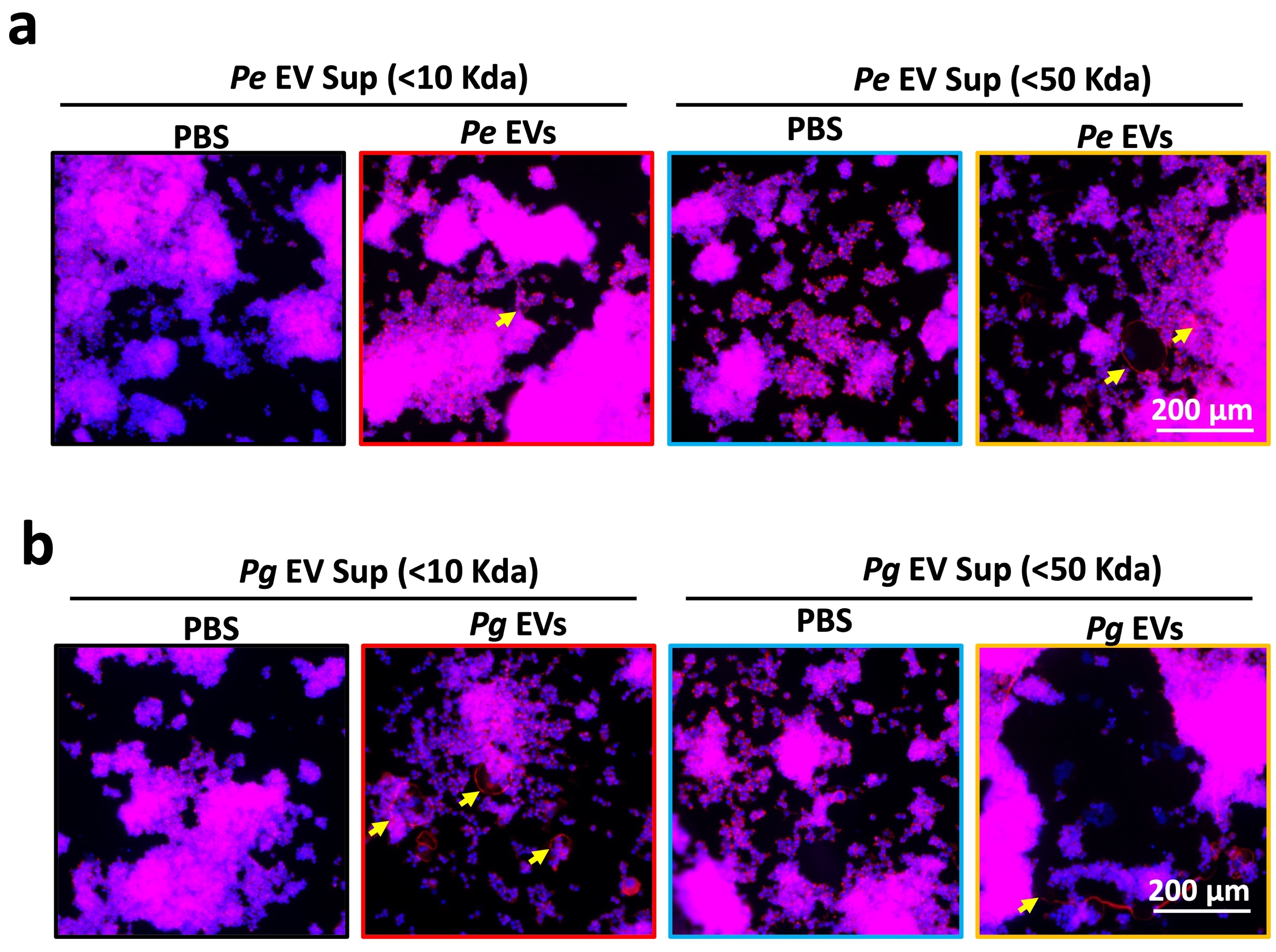
**

**Fig. S14** Newly seeded RAW264.7 cells were cultured in the supernatant (Sup < 50 kDa or Sup < 10 kDa) supplemented with fresh EVs or not for 6 days, after which actin ring staining was performed.

**
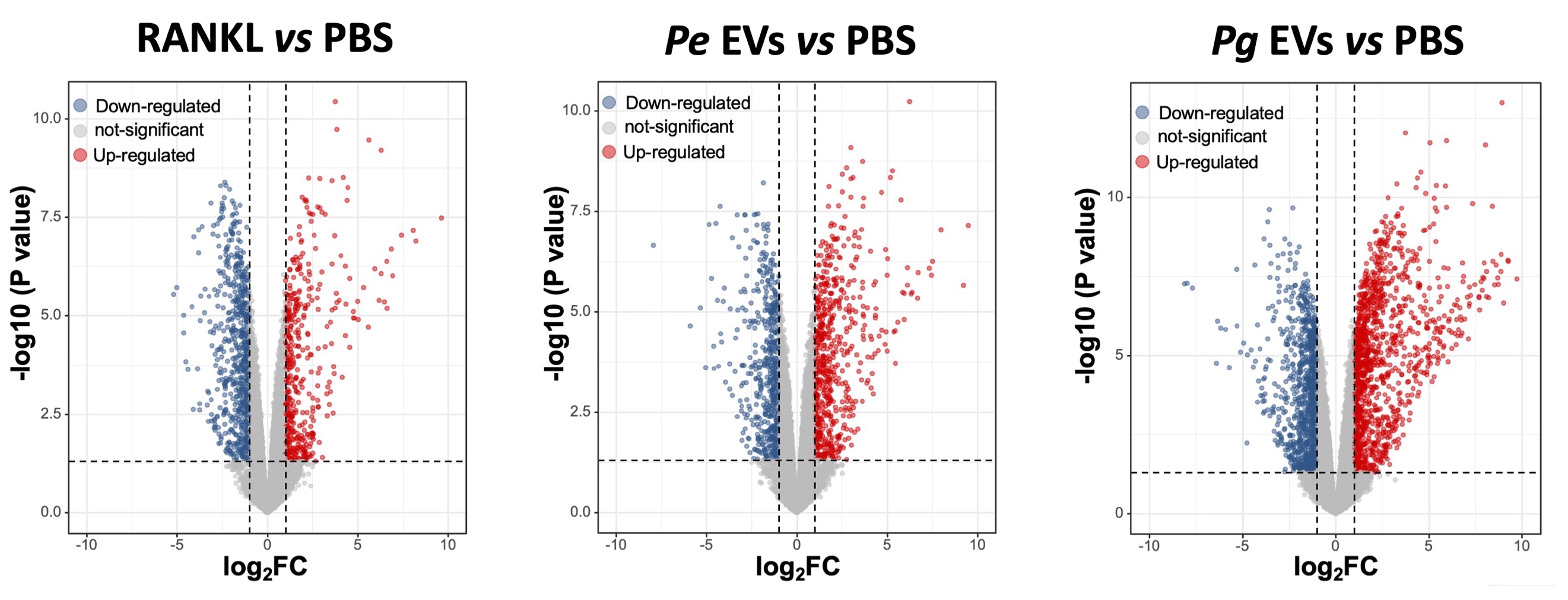
**

**Fig. S15** RNA-seq bioinformatic analysis revealed key differentially expressed genes induced by RANKL and EVs.

**
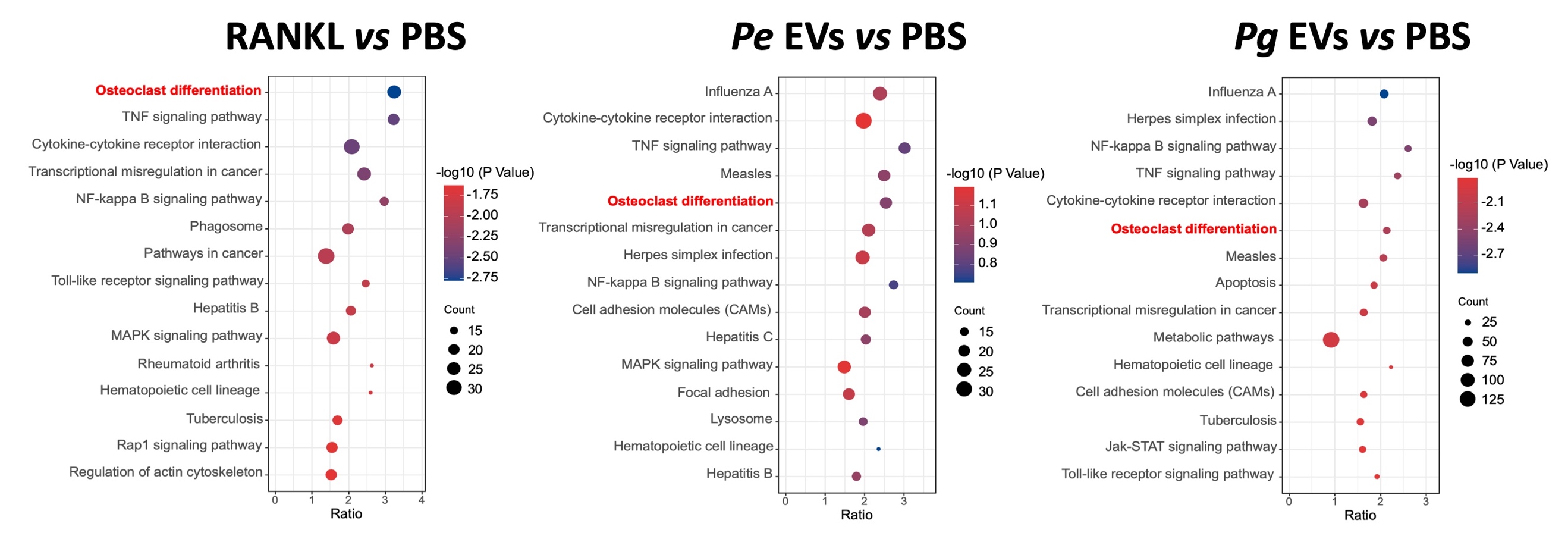
**

**Fig. S16** KEGG pathway enrichment analysis of the top 15 signaling pathways activated by RANKL and EVs.


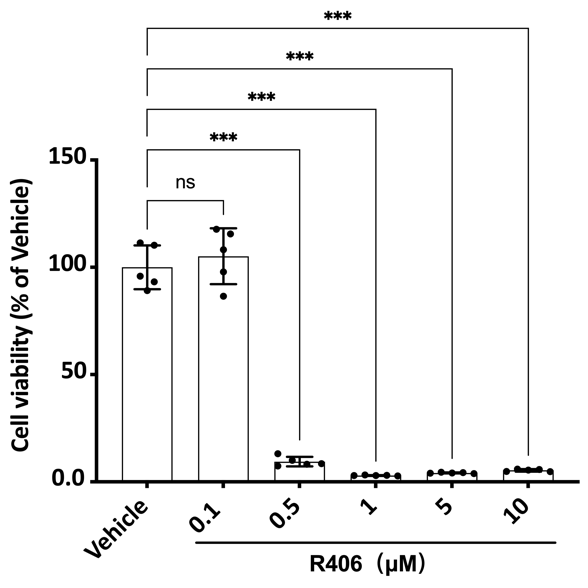


**Fig. S17** CCK-8 analysis was performed to detect the effect of different concentrations of R406 on the viability of RAW264.7 cells. R406 is a highly selective small molecule inhibitor of Syk. ns *P* > 0.05, *** *P* < 0.001.

**
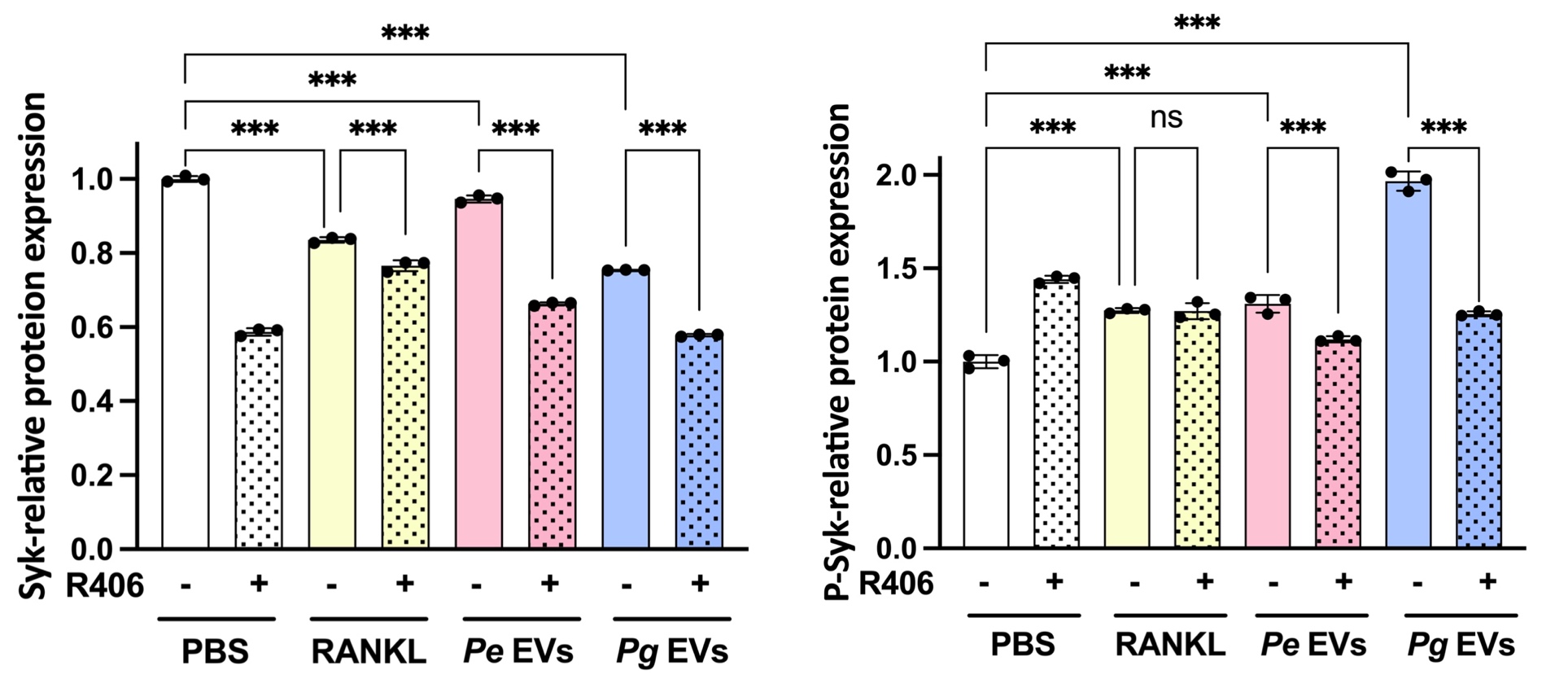
**

**Fig. S18** RAW264.7 cells were treated with RANKL and EVs in the presence or absence of R406 for 24 h, and the relative quantitative analysis of Syk and p-Syk protein expression detected via western blotting was performed using Fiji. ns *P* > 0.05, *** *P* < 0.001.

**
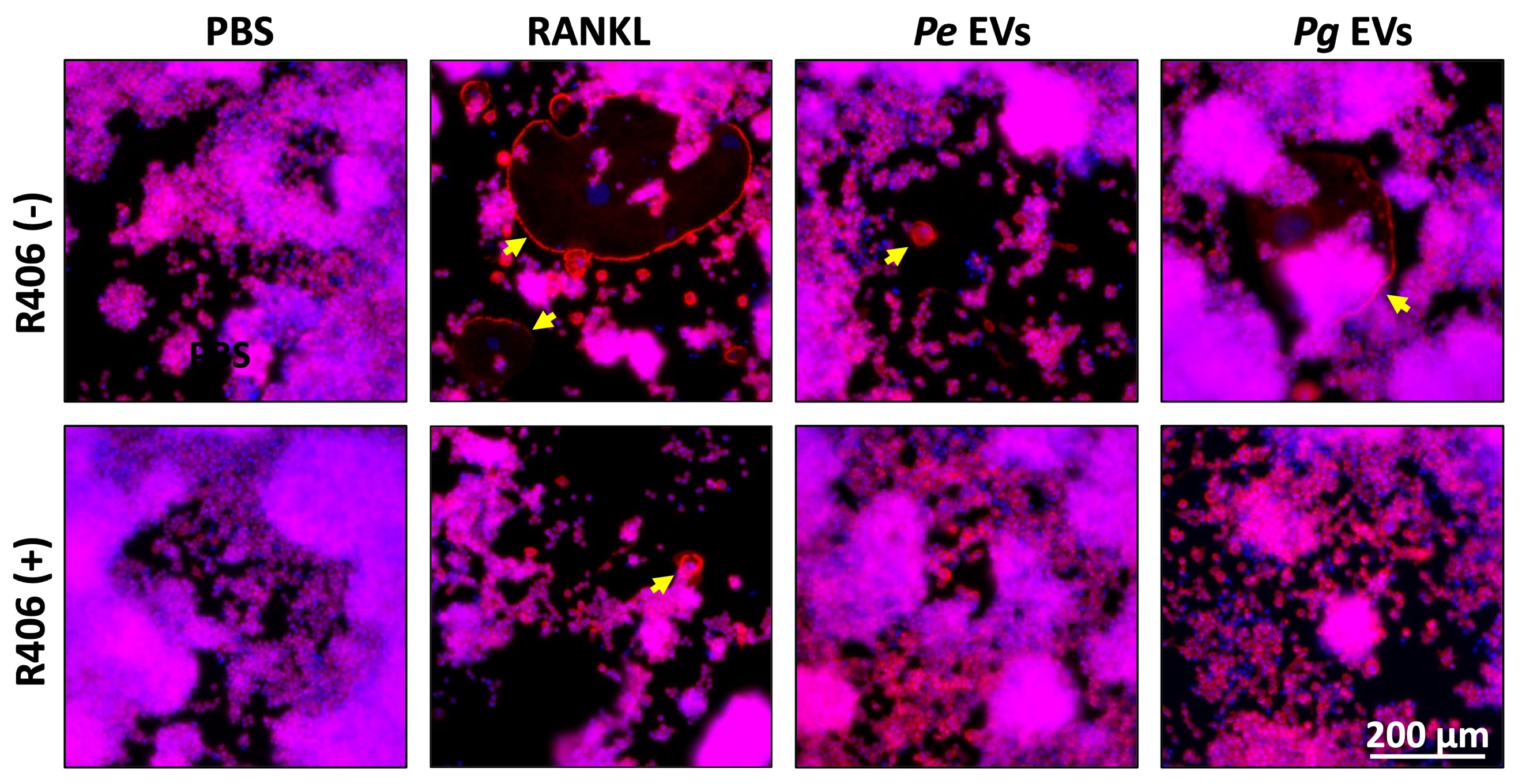
**

**Fig. S19** RAW264.7 cells were treated with RANKL or EVs in the presence or absence of R406 for 6 days, after which actin ring staining was performed.


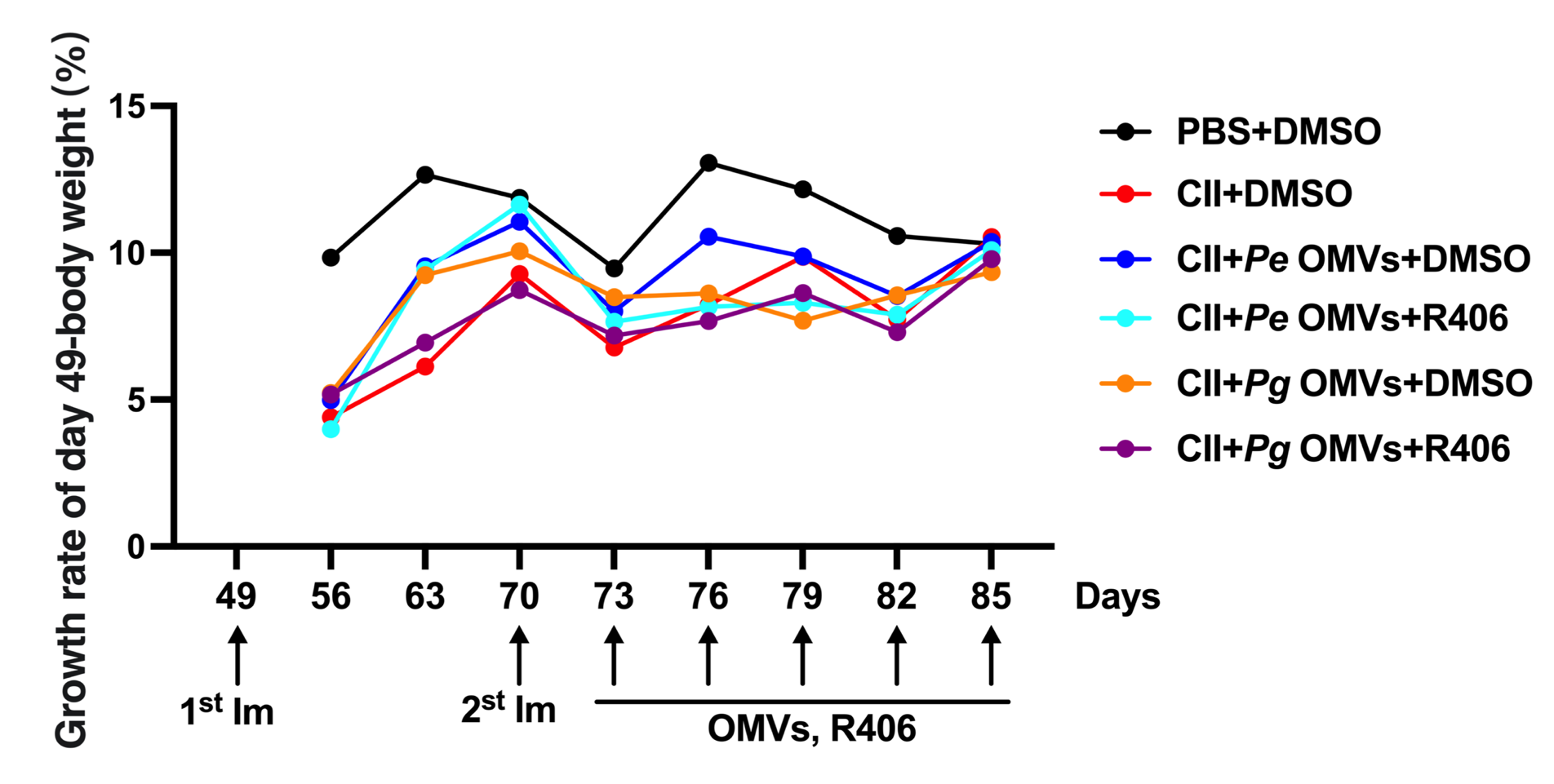


**Fig. S20** The body weight growth rate of the mice. The body weights of the mice were monitored at each time point. The weight growth rate was calculated via the following formula: {weight (day n)/weight (day 49)-1}*100%, where “n” is the observation time point.

**
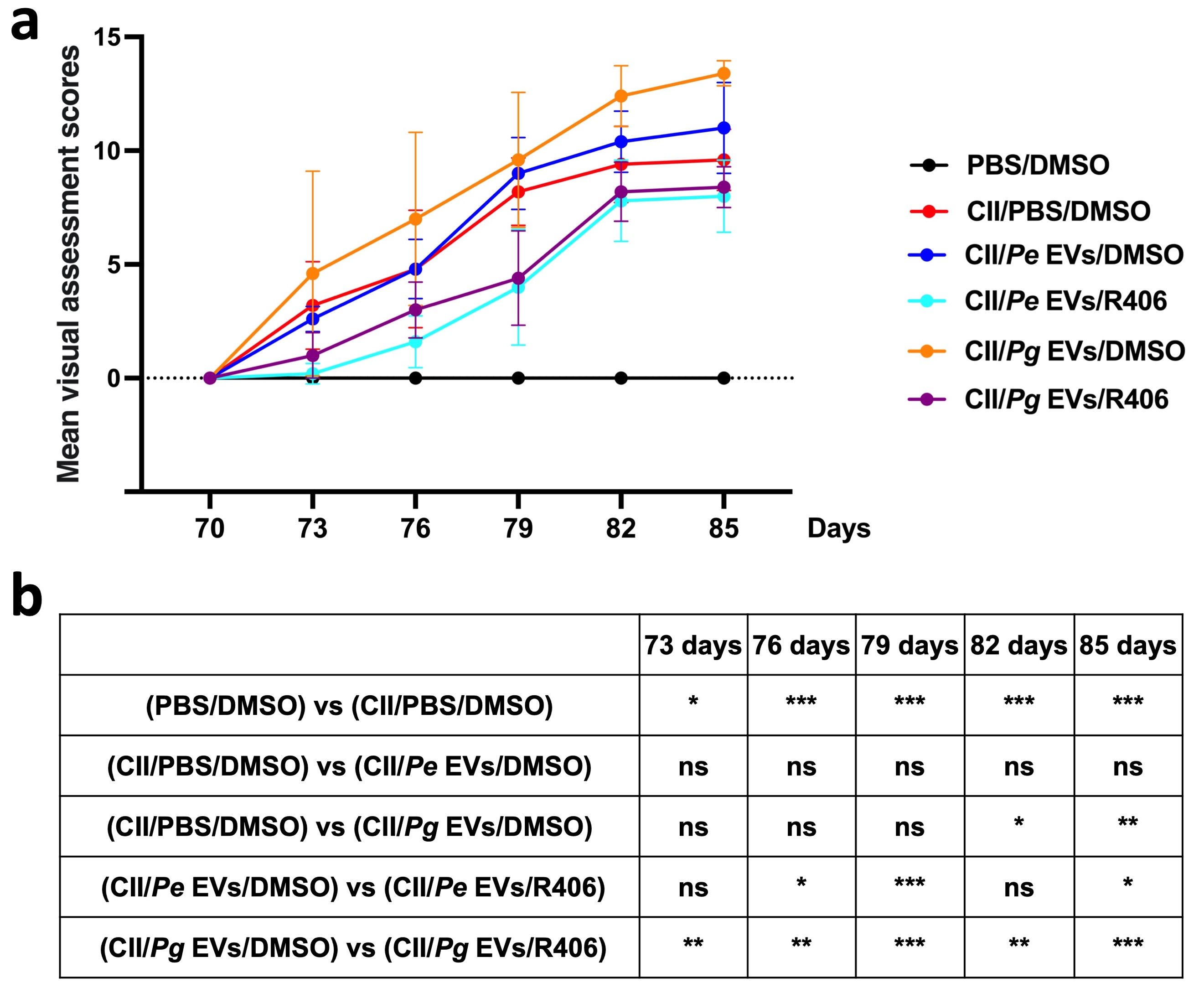
**

**Fig. S21** Mouse Arthritis Score. **a** Arthritis scores of the mice at different observation time points. **b** A comparison of differences among the groups of interest at different time points. ns *P* > 0.05, * *P* < 0.05, ** *P* < 0.01, *** *P* < 0.001.


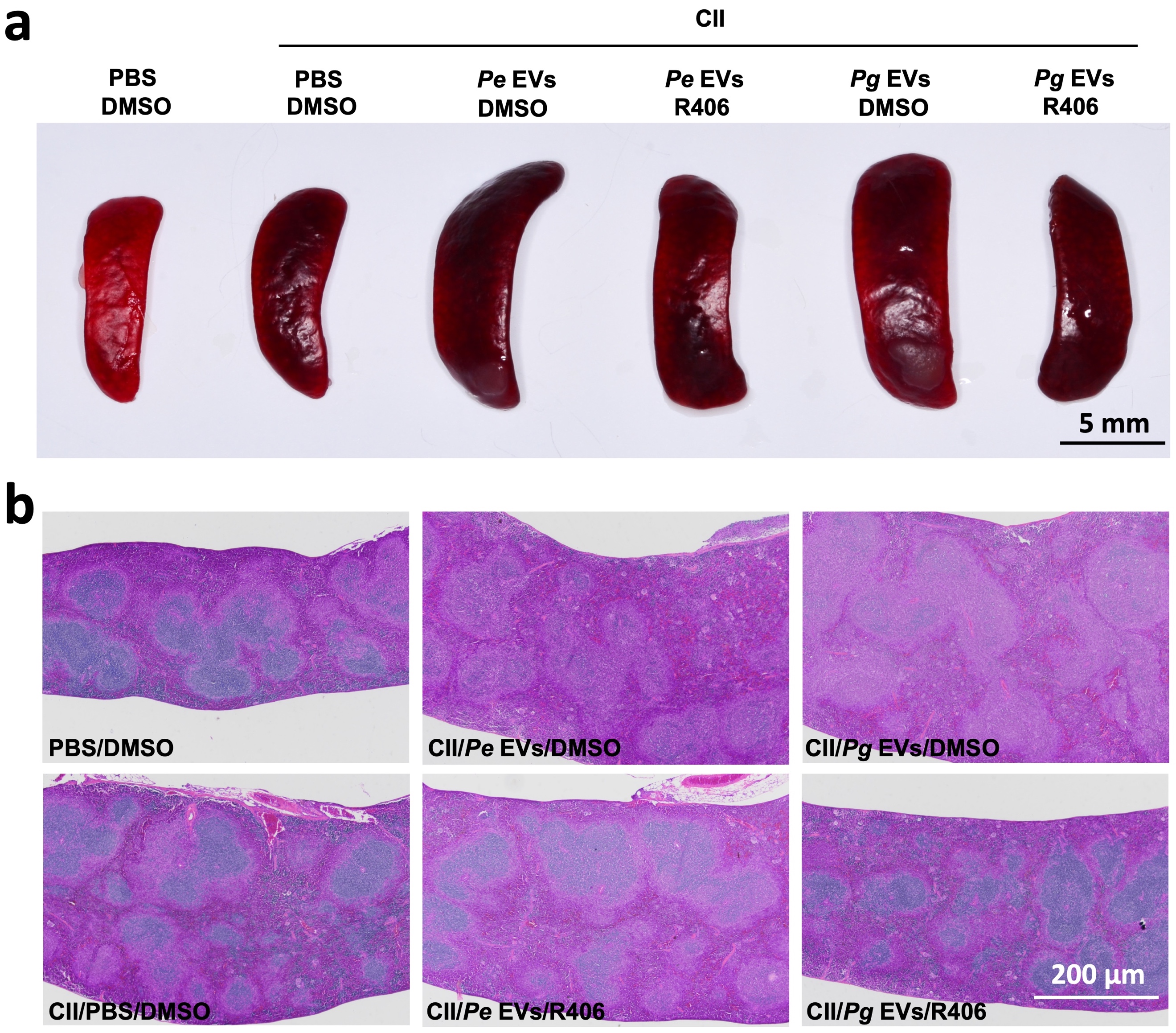


**Fig. S22** R406 alleviated splenomegaly in RA mice exacerbated by EVs. RA mice that received EVs with or without R406 administration. HE-stained mouse spleens sections were observed under a microscope.


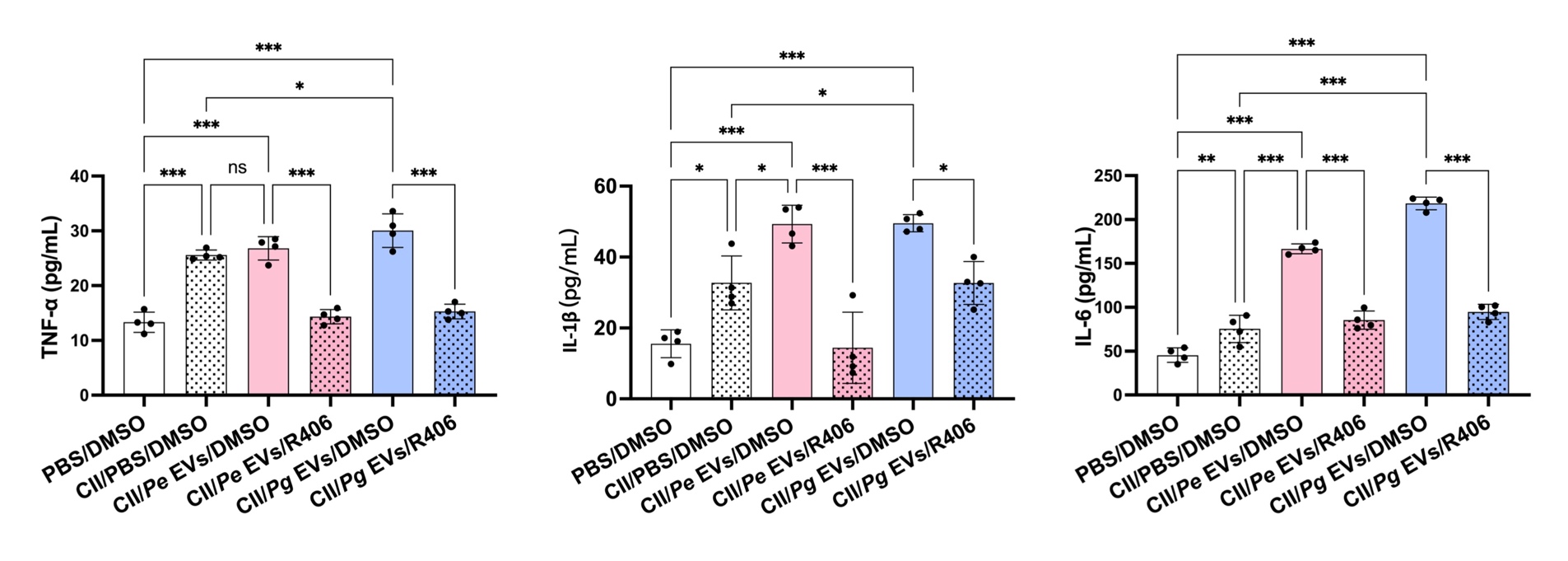


**Fig. S23** R406 attenuated serum inflammatory factor levels induced by *Pg*-EVs in mice with RA. The concentrations of TNF-α, IL-1β, and IL-6 in the serum of the mice were detected via ELISA. ns *P* > 0.05, * *P* < 0.05, ** *P* < 0.01, *** *P* < 0.001.

**
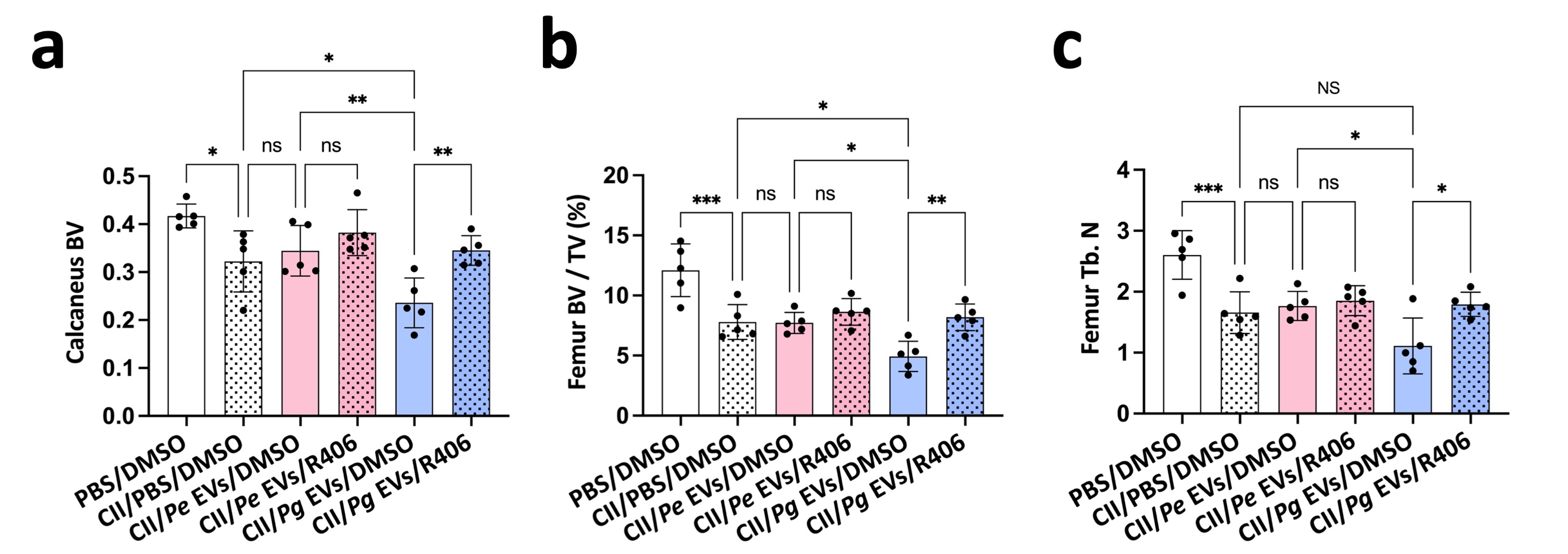
**

**Fig. S24 a** Bone volume (BV) of the calcaneus. **b** Femoral bone volume fraction (BV/TV: bone volume/tissue volume). **c** Femoral bone trabecular number. ns *P* > 0.05, * *P* < 0.05, ** *P* < 0.01, *** *P* < 0.001.


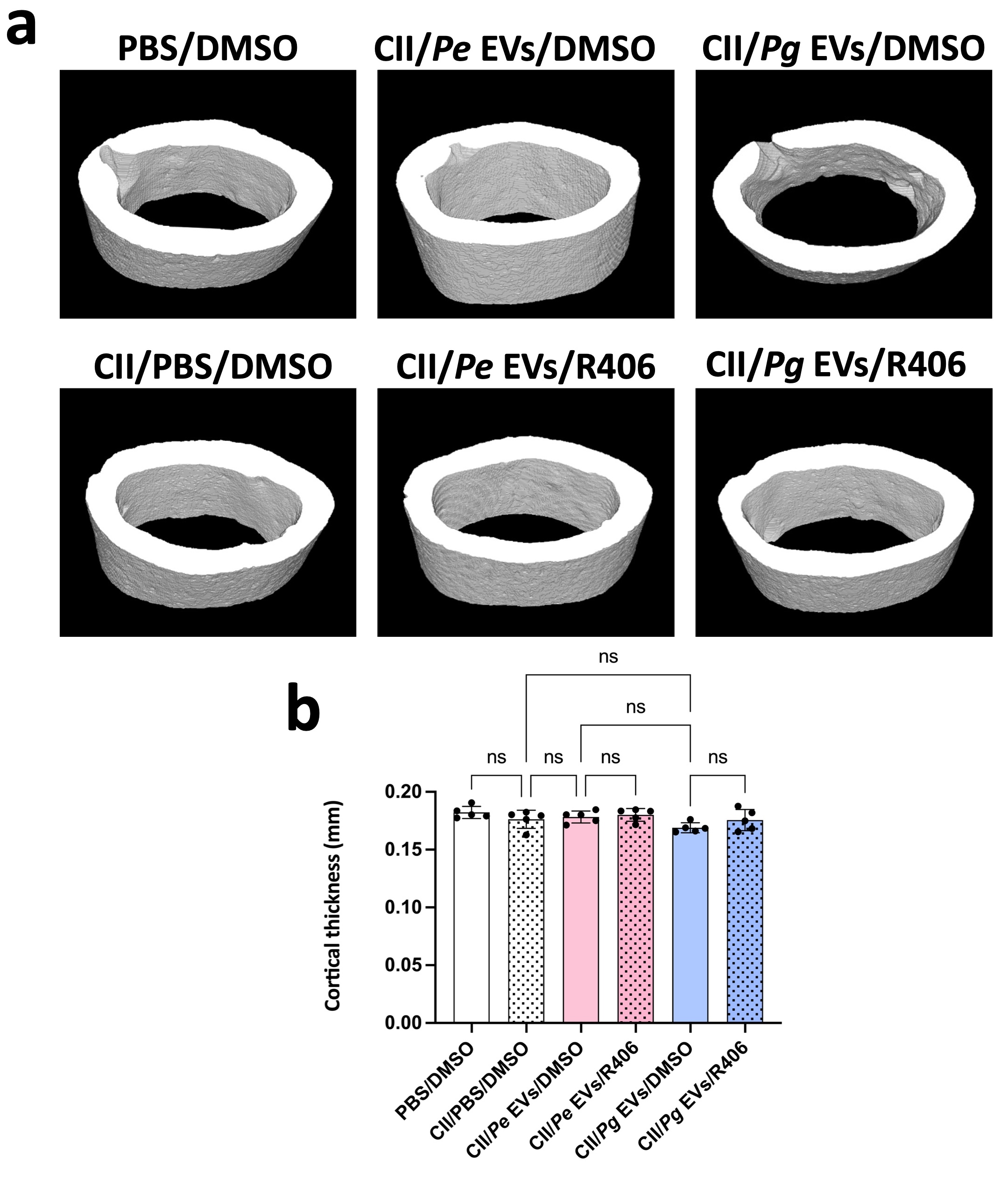


**Fig. S25** EVs did not affect the femoral cortical bone of RA mice. **a** 3D reconstruction photo of the mouse femoral cortical bone. **b** Thickness of cortical bone. ns *P* > 0.05.
